# Transcriptomic Profiling of the Immune Response in Orthotopic Pancreatic Tumours Exposed to Combined Boiling Histotripsy and Oncolytic Reovirus Treatment

**DOI:** 10.3390/pharmaceutics17080949

**Published:** 2025-07-22

**Authors:** Petros Mouratidis, Ricardo C. Ferreira, Selvakumar Anbalagan, Ritika Chauhan, Ian Rivens, Gail ter Haar

**Affiliations:** 1 Joint Department of Physics, Division of Radiotherapy and Imaging, The Institute of Cancer Research, London SM2 5NG, UK; 2 Wellcome Centre for Human Genetics, Nuffield Department of Medicine, University of Oxford, Oxford OX1 2JD, UK; 3 Division of Radiotherapy and Imaging, The Institute of Cancer Research, London SM2 5NG, UK; selvakumar.anbalagan@icr.ac.uk; 4 Research Services, Genomics Facility, The Institute of Cancer Research, London SM2 5NG, UK; ritika.chauhan@icr.ac.uk

**Keywords:** focused ultrasound, therapeutic ultrasound, histotripsy, pancreatic cancer, reovirus, Oncolytic viruses, immunotherapy, transcriptomic profiling, TAP complex

## Abstract

**Background**: Boiling histotripsy (BH) uses high-amplitude, short-pulse focused ultrasound to disrupt tissue mechanically. Oncolytic virotherapy using reovirus has shown modest clinical benefit in pancreatic cancer patients. Here, reovirus and BH were used to treat pancreatic tumours, and their effects on the immune transcriptome of these tumours were characterised. **Methods**: Orthotopic syngeneic murine pancreatic KPC tumours grown in immune-competent subjects, were allocated to control, reovirus, BH and combined BH and reovirus treatment groups. Acoustic cavitation was monitored using a passive broadband cavitation sensor. Treatment effects were assessed histologically with hematoxylin and eosin staining. Single-cell multi-omics combining whole-transcriptome analysis with the expression of surface-expressed immune proteins was used to assess the effects of treatments on tumoural leukocytes. **Results**: Acoustic cavitation was detected in all subjects exposed to BH, causing cellular disruption in tumours 6 h after treatment. Distinct cell clusters were identified in the pancreatic tumours 24 h post-treatment. These included neutrophils and cytotoxic T cells overexpressing genes associated with an N2-like and an exhaustion phenotype, respectively. Reovirus decreased macrophages, and BH decreased regulatory T cells compared to controls. The combined treatments increased neutrophils and the ratio of various immune cells to Treg. All treatments overexpressed genes associated with an innate immune response, while ultrasound treatments downregulated genes associated with the transporter associated with antigen processing (TAP) complex. **Conclusions**: Our results show that the combined BH and reovirus treatments maximise the overexpression of genes associated with the innate immune response compared to that seen with each individual treatment, and illustrate the anti-immune phenotype of key immune cells in the pancreatic tumour microenvironment.

## 1. Introduction

Pancreatic ductal adenocarcinoma (PDAC) presents a formidable treatment challenge due to its exceptionally poor prognosis, low survival, and high disease recurrence rates, leading to approximately half a million deaths worldwide every year [1]. Surgical resection is the current standard of care and is potentially curative, but the proximity of the duodenum and major vessels renders most tumours unresectable. For patients with unresectable PDAC, first-line treatments include the administration of FOLFIRINOX (folinic acid, fluorouracil, irinotecan, and oxaliplatin) [2,3], and, in a metastatic setting, a combination of gemcitabine and nanoparticle albumin-bound paclitaxel is beneficial [4]. However PDAC tumours generally respond poorly to chemotherapy, and in most cases, survival is measured in months rather than years. The application of immunotherapy has yet to demonstrate therapeutic benefits for PDAC patients. This lack of efficacy is due to resistive mechanisms, which include a dense stroma that impedes drug penetration [5,6], and an immune-suppressive tumour microenvironment (TME) [7]. There is therefore a pressing need for adjuvant approaches that could help to overcome the immune resistance of immunologically “cold” PDACs.

Oncolytic viruses (OVs) provide a form of immunotherapy and are known to modulate the TME in tumours stratified as ‘less immunosuppressive’ [8]. These viruses have natural oncolytic properties, being non-pathogenic and replication-competent, and infect cancer cells either through endocytosis or by binding to virus entry surface receptors. OVs trigger a cell’s demise by inducing lysis and immunogenic cell death and are subsequently eliminated by the adaptive immune response [9,10]. The oncolytic Reovirus is being investigated for the treatment of pancreatic cancer, but to date with only limited success. The NCT01280058 phase II ‘Carboplatin and paclitaxel with or without viral therapy in treating patients with recurrent or metastatic pancreatic cancer’ trial [11] investigated the unmodified reovirus type 3 Dearing (Pelareorep) in combination with chemotherapy, with the control arm receiving chemotherapy alone (*n* = 37). This trial found no significant difference in the progression-free survival between the two groups, although increases were seen in pro-inflammatory immune biomarkers (T and natural killer (NK) cells, cytokines/chemokines) [12] in the combined reovirus and chemotherapy-treated arm relative to the control arm. In another phase II trial (NCT00998322; A study of REOLYSIN^®^ in combination with gemcitabine in patients with advanced pancreatic adenocarcinoma) 23 out of 34 patients had stable disease following the combined treatment which was well tolerated [13]. A phase I clinical study (NCT03723915; Pembrolizumab and Pelareorep (reovirus) in treating patients with advanced pancreatic cancer) showed significant increases in circulating immune cells and various pro-inflammatory chemokines and cytokines when reovirus was present [14,15]. These results, and the requirement to increase the PDAC infection rates [13], demonstrate the potential for the use of adjuvant therapies, in combination with reovirus, to tap the immense potential for increasing immunogenic PDAC cell death.

Focused ultrasound is a physical modality which can destroy tumours whilst sparing normal tissue [16]. It is a rapidly expanding therapeutic field, and the full “gamut” of its applications can be seen in a report from the Focused Ultrasound Foundation [17]. Focused ultrasound can induce either thermal and/or mechanical effects inside the target tissue and can be switched from predominantly thermal to predominantly mechanical effects by changing the mode of energy delivery [18]. Boiling histotripsy (BH), one of the “mechanical” modes of focused ultrasound tissue damage, induces acoustic cavitation (clouds of highly energetic micron-sized bubbles) which initiates mechanical cellular disruption via bubble collapse [19]. Clinical trials of focused ultrasound-created thermal ablation in PDAC tumours have demonstrated feasibility and safety [20], whereas phase I histotripsy trials for liver, pancreatic and kidney cancers are underway [21,22].

Our group has demonstrated that “mechanical” focused ultrasound using exposures in the BH range [23] (peak negative pressure (P-) ~17 MPa, duty cycle (d.c.) 1%, pulse repetition frequency (prf) 1 Hz, pulse length 10 msec, lesion spacing = 2 mm) in combination with checkpoint inhibitor immunotherapy (anti-CTLA-4 and anti-PD-1 antibodies) can induce anti-cancer immune effects in PDAC tumours by disrupting the tumour core and changing the immune architecture of the tumour to a pro-inflammatory phenotype [24]. Despite the beneficial effects of these treatments, viable tumours remained, and ultimately the subjects succumbed to their disease. Here, we extend our previous study to investigate the acute immune response of murine PDAC tumours exposed to BH treatments using single-cell transcriptomics to elucidate the gene modulation of a variety of immune cell types. To enhance the immune response of these “immune-cold” PDAC tumours, ultrasound treatments were combined with reovirus as an immune adjuvant. We hypothesise that the two treatments, when combined, will show greater immune cell and gene modulation than either of the two treatments alone, and that this will help us better understand the regulation of the various immune cell types and mechanisms associated with the BH treatment response in PDACs.

## 2. Materials and Methods

Orthotopic PDAC murine model: Murine subjects were purchased from Charles River (UK), and housed in semi-sterile conditions in the animal facilities of the Institute of Cancer Research. Experimental work was carried out under the relevant UK Home Office project and personal licences. Ethics approval was obtained from the local Animal Welfare and Ethics Committee of The Institute of Cancer Research, London. The subjects were monitored regularly for their quality of life (including weight, condition, behaviour, and pain) to ensure the highest welfare standards for the subjects. Their Body Condition Score, the NC3R Grimace Scale, and the modified FELASA Clinical Signs were used to ensure that no animal exceeded the permitted severity limit. Subjects were sacrificed using Schedule 1 humane methods.

The pancreatic KPC cell line (*KRASG12D/+*; *TRP53R172H/+*; *PDX-1-CRE*) of low passage (<5) (kindly provided by Prof. Tuveson (CSHL, NY, USA)) was cultured in Dulbecco’s modified eagle medium supplemented with 2 mM L-glutamine, and 10% foetal bovine serum. All reagents used for cell culture were purchased from Sigma-Aldrich (Gillingham, UK) unless otherwise stated. KPC cells were short tandem repeat (STR) profiled, and routinely tested for mycoplasma. KPCs grown in T75 flasks were harvested using Accutase and 10 × 10^6^ cells/mL were resuspended in Engelbreth–Holm–Swarm murine sarcoma gel. Syngeneic KPC tumours were grown orthotopically by injection of 20 μL of the above cell suspension into the pancreas of C57BL/6 (strain 000064J) mice via a laparotomy (Appendix A). Tumour growth was monitored once a week using the E-Cube 9 (Alpinion Medical Systems, Bothell, WA, USA) 2-D B-mode ultrasound imaging system (central frequency = 14 MHz). PDACs were imaged every 1 mm in the cranial/caudal (sagittal) and medial/lateral (axial) imaging planes. The maximum dimension in 3 orthogonal directions was measured. Typically, tumours could be treated 4 weeks post KPC cell implantation, once all orthogonal dimensions were approximately 7 ± 2 mm.

Experimental studies: For single-cell transcriptomic analysis 24 h post “treatment”, subjects (*n* = 21) were randomised into four groups—Group 1: sham-exposed, “control” (*n* = 6); Group 2: reovirus-treated (*n* = 6); Group 3: BH-exposed (*n* = 5); Group 4: BH and reovirus “combination” treatments (*n* = 4). We intended to allocate at least 5 subjects per treatment group; one sample from group 4 (combination) was unfortunately lost during sample processing. For histology experiments 6 and 72 h post “treatment”, 2 subjects were used as controls, and 3 subjects were used for every other group at each time point. For both histology and single-cell transcriptomic experiments, time zero hours is defined as the end of a subject’s “treatment” with BH or sham-BH exposures.

**Figure A1:**
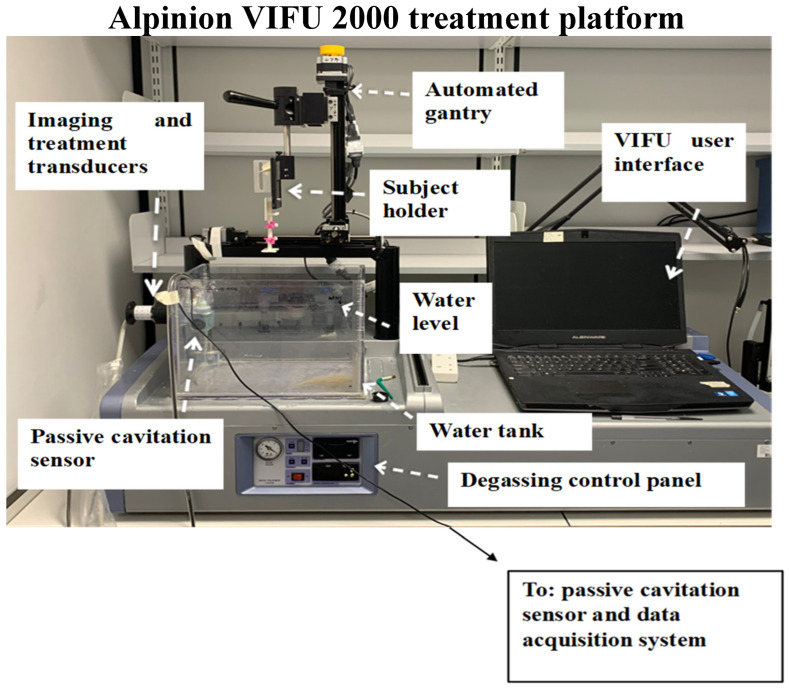
The Alpinion VIFU 2000 treatment platform used to treat murine subjects with boiling histotripsy.

BH exposures (groups 3 and 4) were carried out using the Alpinion VIFU 2000 therapy ultrasound platform (Alpinion Medical Systems, Bothell, WA, USA) (Figure A1). The water tank and subjects were prepared as described in Appendix A. The positions of the PDAC tumour’s upper and lower edges were identified using the 3–12 MHz US imaging probe of the VIFU 2000 system. Subsequently, the central regions of the tumour were located and exposed under ultrasound imaging guidance to avoid damage to adjacent organs/tissues at risk (spleen, stomach, intestine, peritoneum, skin, and abdominal wall). To achieve this, no exposures were placed within ~2 mm of the tumour edges. The number of treated positions per tumour (10 to 30) was dependent on tumour size, and on how well it could be differentiated from its surroundings. Also, to achieve the safe delivery of ultrasound energy in every tumour, every time significant hyperechoic ultrasound signals were seen using real-time ultrasound imaging during exposure, the exposures were immediately stopped, and the treatment was moved to the next planned position. This is because, in our experience, over-treatment of murine orthotopic tumours with BH can have detrimental quality-of-life effects.

The parameters used for BH exposures of each position were 10 msec pulses at a frequency of 1.5 MHz, d.c. 1%, prf 1 Hz, with a spacing between treatment planes and exposure positions in each plane of 1 mm. The free-field P^−^ (~17–19 MPa), electrical power (200–225 W), and number of exposure pulses delivered per position (up to a maximum of 25 and at least 12) varied slightly depending on the bright hyperechoics seen on real-time ultrasound imaging feedback during exposure. Calibration of the VIFU2000 treatment transducer was performed by using a calibrated hydrophone to obtain free-field pressure values as a function of the system’s electrical power settings at low levels (<−5 MPa) to avoid sensor damage and extrapolated to higher levels. Subjects in groups 1 and 2 were sham-exposed by depilating the anaesthetised subjects, and placing them in the VIFU’s heated water bath for 10 min in a similar manner to groups 3 and 4, but without treating them with BH.

For oncolytic reovirus treatments, stocks of T3Dearing strain reovirus were obtained from Oncolytics Biotech Inc, and aliquots of ~3.2 × 10^9^ pfu/mL were banked at −80 °C for long-term storage [25,26]. Mice were incubated at 37 °C for 10 min before reovirus was administered systemically through an IV tail injection in 3.2 × 10^7^ pfu/dose in Hanks balanced salt solution (HBSS), for groups 2 and 4, within 30 min post-BH treatment. Subjects in groups 1 and 3 were sham-exposed using IV tail injections of the vehicle only.

Acoustic cavitation detection and data processing: Acoustic cavitation was monitored in a semi-quantitative manner using a passive cavitation detector (PCD) and a hardware filter which removed the 1.5 MHz drive frequency, as described previously [16,24]. Briefly, the PCD was pulse-echo aligned manually with the BH focus at the beginning of each experimental day (in the absence of the electrical filter). Acoustic emission data were recorded for each pulse, and processed using custom-made MATLAB scripts (version R2020b) which quantified the half-harmonic (0.75 MHz) and broadband signals in the range 0.1 to 3 MHz, following removal of all drive harmonics and subharmonics using a software comb filter. This broadband range was chosen to minimise the detection of imaging signals due to using imaging to detect hyperechoes during exposures. Every PCD detected exposure pulse was divided into 120 cycle segments and fast Fourier transformed to obtain a series of emission spectra. The broadband signal for each exposure pulse was quantified as the sum of voltage amplitudes at each frequency within the broadband range after software (comb filter) removal of harmonic and ultraharmonic signals. Half-harmonic signal for each exposure pulse was the sum of voltages in 3 frequency bins centred on the half-harmonic. Each pulse was characterised using the median amplitude of the half-harmonic and the comb-filtered broadband from all segments. If at least 3 consecutive segment amplitudes were above baseline noise (the average “off-time” signal plus 3 standard deviations) the pulse was identified as a positive (half-harmonic or broadband) event. For each tumour, the total number of exposure pulses and half-harmonic-positive and broadband-positive pulses were calculated. Then the percentage of half-harmonic- and broadband-positive exposure pulses was computed

Histology: For histological analysis, the subjects were culled 6 or 72 h after treatments. The 6 h time point was chosen because the histological effects of BH are likely to be immediate. The 72 h time point was chosen as a reasonable indicator of the delayed tissue effects of BH exposures. The excised KPC tumours were fixed in 10% neutral buffered formalin for 48 h and processed for paraffin embedding overnight. Sections with a thickness of 5 μm were then cut using a microtome, and placed on adhesive slides coated with poly-lysine. These slides were subsequently incubated for 30 min on a 55 °C hot plate, and stored at room temperature. Haematoxylin and eosin (H&E) staining was carried out as described in Appendix A.

Cell preparation and cell sorting for multi-omics experiments: For single-cell experiments, KPC tumours were disintegrated 24 h after treatment using the gentleMACS™ Octo dissociator with Heaters kit and processed for single-cell suspension following the manufacturer’s protocol (Miltenyi Biotech, Bergisch Gladbach, Germany), briefly described in Appendix A. Then, single-cell multi-omics was performed using the BD Rapsody system, combining whole-transcriptome profiling (WTA kit; BD Biosciences, San Jose, CA, USA) with the expression of surface-expressed proteins using the AbSeq technology (BD biosciences). To profile the changes of treatment on the immune compartment, CD45^+^ cells (e.g., T and B lymphocytes, monocytes/macrophages, dendritic cells, and granulocytes, including neutrophils) were isolated and profiled using the BD Rhapsody HT Xpress system (BD Biosciences). Briefly, cell-containing frozen vials were thawed in a water bath at 37 °C and agitated until only a small ice clump remained. X-Vivo medium (1 mL) supplemented with 1% heat-inactivated FBS was then added to each vial in a brisk, drop-by-drop manner. The contents of each vial were transferred into a sterile pre-labelled 15 mL centrifuge tube, made up to 10 mL using additional X-Vivo 15 (Lonza, Basel, Switzerland) medium supplemented with 1% FBS, and mixed by gently swirling, followed by centrifugation at 400× *g*, for 7 min, at room temperature to pellet the cells. The supernatant was carefully discarded, and 500 mL of ACK lysis buffer was added to the cell pellet and mixed for 1 min. This mixture was made up to 13 mL final volume with ice-cold PBS + 2% FBS and was centrifuged at 400× *g*, 7 min, 4 °C. The cell pellet was resuspended in 5 mL of cold PBS + 2% FBS. Cells were counted and distributed into 10^6^ cell aliquots. Each of the aliquots was transferred into a sterile pre-labelled FACS tube and topped up with ice-cold PBS + 2% FBS. Tubes were centrifuged at 400× *g*, 5 min, 4 °C. Pellets obtained were resuspended in 50 mL of BD FACS buffer. Then 2 mL mouse Fc block (1 μg/million cells) was added and incubated on ice for 5 min. Cell aliquots were then labelled with the respective sample barcoding antibody (sample multiplexing kit; BD Biosciences) and with FITC-conjugated anti-mouse CD45 antibody (BioLegend, San Diego, CA, USA) for a further 15 min on ice. 110 μL BD FACS buffer was then added to the tubes (BD biosciences) and cells were stained with DRAQ7 (1:500 dilution) for 15 min on ice for the identification of dead cells. Cells were then washed and resuspended in cold PBS + 1% FBS and filtered for cell sorting at 4 °C in a BD FACSAria Fusion sorter (BD Biosciences). A total of 5 × 10^4^ CD45^+^ DRAQ7^−^ live cells were sorted from each experimental condition. Cells were processed into three experimental days, with 8 samples (duplicates of each of the 4 experimental conditions) being processed on each day.

AbSeq antibody stain and single-cell capture: After cell sorting, barcoded cells from 8 samples (2 samples per experimental condition) were pooled together and incubated with a master mix of oligo-conjugated AbSeq antibodies (BD Bioscience; as listed in Table A1) for 35 min on ice, according to the manufacturer’s instructions. Cells were then washed three times with BD Sample Buffer at 4 °C to remove residual unbound oligo-conjugated AbSeq antibodies, resuspended in 500 mL cold BD Sample buffer, and filtered for cell counting. Three sample aliquots were then resuspended in 620 mL of cold BD sample buffer at a concentration of 40 cells/mL—for an estimated capture rate of ~15,000 single-cells/cartridge—and immediately loaded on three BD Rhapsody cartridges (BD Biosciences) for single-cell capture.

Transcriptomic data analysis: Fastq files were processed using the Institute of Cancer Research London installation of BD Rhapsody Sequence Analysis Pipeline (v1.11) to generate expression count matrices for RNA and Abseq data, which were analysed in R using the Seurat package (v4.3.0). Quality control was performed separately for data from the three BD Rhapsody cartridges, filtering out cells with fewer than 300 or more than 5000 detected genes, fewer than 500 unique molecular identifier (UMI) counts, or greater than 20% mitochondrial content. Genes detected in fewer than 10 cells were also excluded. After quality control, the filtered datasets were merged for downstream analysis. RNA and Abseq data were normalised separately, with RNA processed using the LogNormalize function and Abseq data normalised using the centred log-ratio (CLR) method. Batch correction and integration of Abseq data were performed using the reciprocal PCA (RPCA) method, followed by feature selection using FindVariableFeatures and data scaling with ScaleData. Dimensionality reduction was performed using PCA on RNA data and significant principal components were selected based on the elbow plot method. To integrate RNA and Abseq modalities, a Weighted Nearest Neighbour (WNN) graph was constructed using FindMultiModalNeighbours. Clustering was performed using the Louvain algorithm (FindClusters) at a resolution of 0.9, to detect distinct immune cell subsets within the TME of pancreatic tumours across all subjects included in the study and clusters visualised using the Uniform Manifold Approximation and Projection (UMAP). FindConservedMarkers was used to identify the top markers for each cluster that were conserved between the four treatment conditions. Cell-type annotations were manually assigned based on established markers and the literature. Pseudobulk differential expression analysis was performed in R using the DESeq2 package, and gene ontology enrichment analysis was conducted with the clusterProfiler R package to evaluate treatment effects on immune cell subpopulations and their associated biological processes.

Statistical analysis: Immune cell abundance and immune cell/Treg ratio data are shown as mean ± standard error of the mean (SEM). The number of experimental replicates is stated in each figure and statistical significance is calculated using a 2-way unpaired equal variance Student’s T test. The statistical significance of differences in transcriptomic data is automatically calculated using *p*-adjusted values in R (version 4.3.1). In all cases, differences were considered statistically significant at *p* < 0.05. Unless otherwise stated in the manuscript, gene overexpression was considered “high” if their AvgLog_2_Fc > 1, “moderate” if 1 ≥ AvgLog_2_Fc ≥ 0.5, “low” if 0.5 > AvgLog_2_FC > 0.25, and “negative” if AvgLog_2_FC < 0.25.

## 3. Results

### 3.1. BH Treatment Monitoring and Acoustic Cavitation Monitoring

BH exposure of tumours resulted in the detection of half-harmonic-positive and broadband-positive signals. In the BH alone group, 17–30 positions/tumour were exposed to BH (on average, 24 ± 6), and 350–720 pulses per tumour were delivered (on average, 550 ± 150). At least 98% of these pulses were positive for half-harmonic signals, and the number of half-harmonic-positive pulses per tumour ranged between 350 and 720 (Table 1). In the same treatment group, the percentage of BH pulses resulting in broadband signals ranged between 14 and 94% per tumour, and the number of broadband-positive pulses ranged from 95 to 360 per tumour (Table 1). In the combination group, 10–22 positions/tumour were exposed to BH (on average, 15 ± 5), and 230–530 exposure pulses per tumour were delivered (on average, 350 ± 130). Here, more than 91% of the pulses were positive for half-harmonic signals, and the number of half-harmonic-positive pulses per tumour ranged from 210 to 530 pulses. Also, the percentage of broadband-positive pulses ranged from 2 to 100%, and the number ranged from 6 to 310 per tumour (Table 1).

**Figure 1:**
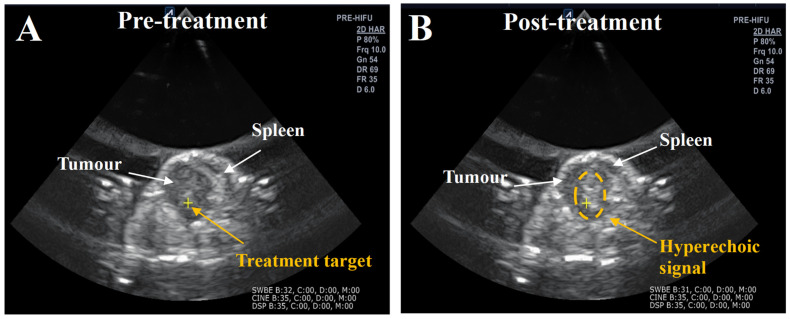
Examples of ultrasound images of BH-treated KPC tumours immediately before and after treatment. Ultrasound images of subject 3 (Table 1-combination group) before (**A**) and after (**B**) the BH treatment. Before treatment, the tumour has a round shape, is less echogenic than its surroundings, and can be readily distinguished from the spleen. The yellow cross denotes a treatment target position. Immediately after treatment, a bright hyperechoic signal is typically seen within the tumour, indicated by the amber circle and arrow, which acts as an independent indicator for the induction of acoustic cavitation in the tumour.

Treatment of the KPC tumours with BH resulted in the formation of hyperechoic regions seen using ultrasound imaging in all except one tumour. These hyperechoic signals were seen in the core of the tumours where the exposures were targeted, and no such signals were detected outside the tumours (Figure 1). Treatment delivery resulted in no side effects such as decreased quality of life, signs of pain, or the development of damage to the overlying skin, peritoneum, or other adjacent organs in subjects as assessed at the time of dissection. Despite these treatments, all tumours (control, reovirus, BH, combination groups) continued to grow, with the subjects ultimately succumbing to their disease.

### 3.2. Histological Analysis Following Treatments

**Figure 2:**
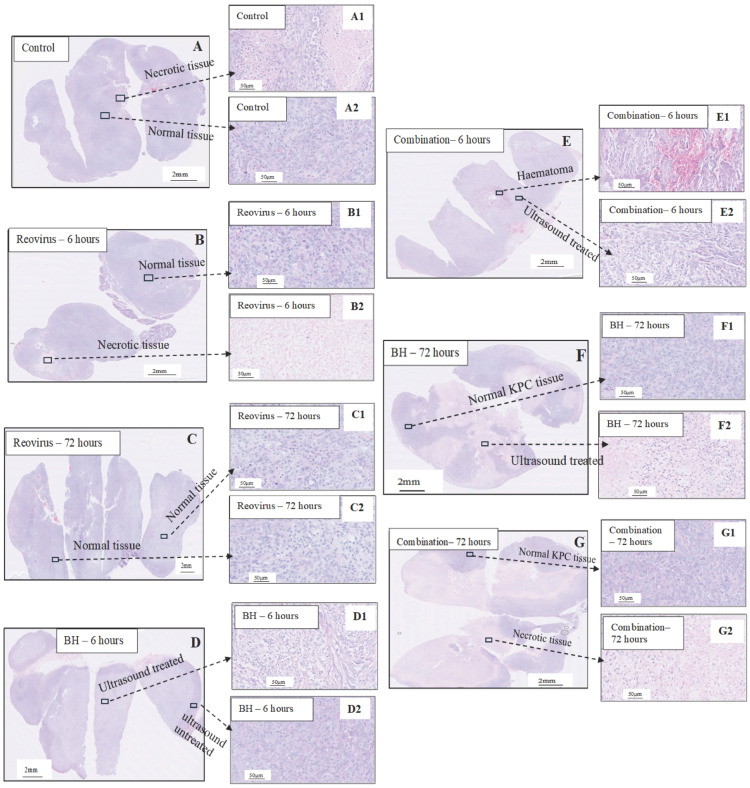
H&E staining of PDAC tumours 6 and 72 h after treatments. KPC tumours were excised 6 or 72 h after treatment with reovirus (**B**,**C**), BH (**D**,**F**), or combined BH + reovirus (**E**,**G**). Cell nuclei stain purple (haematoxylin), while cytoplasm and fibres stain pink (eosin). A sham-exposed tumour is shown in (**A**). The sham and reovirus treated tumours contain densely packed cells (**A2**,**B1**,**C1**,**C2**) and small areas of necrosis devoid of cells (**A1**,**B2**). Tissues exposed to BH and BH + reovirus show extensive morphological changes 6 h after treatment, characterised by increased intercellular spaces (**D1** and **E2**, respectively). Also, in this example of BH + reovirus treatment, red-stained haematomas were seen (**E1**). Occasionally haematomas were also seen in the sham-exposed tumours (**A**). 72 h after BH or BH + reovirus treatment extensive necrotic areas had developed (**F2**,**G2**). Finally, the peripheral parts of ultrasound-exposed tissues showed normal tissue moprhopology that consisted of densely packed cells (**D2**,**F1**,**G1**).

Sham-exposed and reovirus-treated tumours had densely packed KPC cells covering their core and periphery 6 and 72 h after treatment (Figure 2A–C). Occasionally, necrotic-looking areas characterised by a reduced number of cell nuclei (Figure 2A1,B2) and haematomas (Figure 2A) could be seen. When tumours were exposed to BH, either alone or in the combination group, extensive tissue disruption was seen 6 h after treatment (Figure 2D and Figure 2E, respectively). These ultrasound-treated tissues exhibited morphological tissue structure features including increased intercellular gaps, and abnormal-looking and elongated nuclei (Figure 2D1,E2). In some cases, relatively large haematomas were observed (Figure 2E1). Seventy-two (72) hours after combination and BH-only exposures, extensive necrotic-like regions were seen (Figure 2F,G). These areas had markedly different nuclear and cytoplasmic staining compared to the sham-exposed and reovirus-treated tissues, as well as compared to the normal-looking KPC peripheral tissue of the same tumours (Figure 2F1,G1). These differences included eosin-stained intercellular gaps possibly consisting of debris and fibres in the treated areas and a low number of cell nuclei (Figure 2F2,G2).

### 3.3. Characterisation of Immune Subset Populations

**Figure 3:**
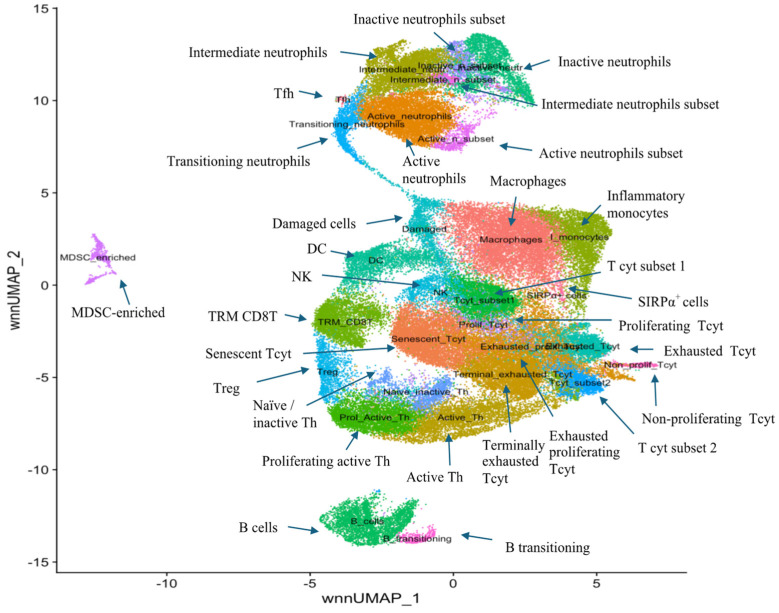
The UMAP of KPC tumours used in this study. The two-dimensional gene expression UMAP showing 30 immune cell clusters identified from gene expression analysis obtained from all the KPC tumours (control and treated) and manually annotated using the biomarkers shown in Table 2, Table A2 and Table A3.

**Figure A2:**
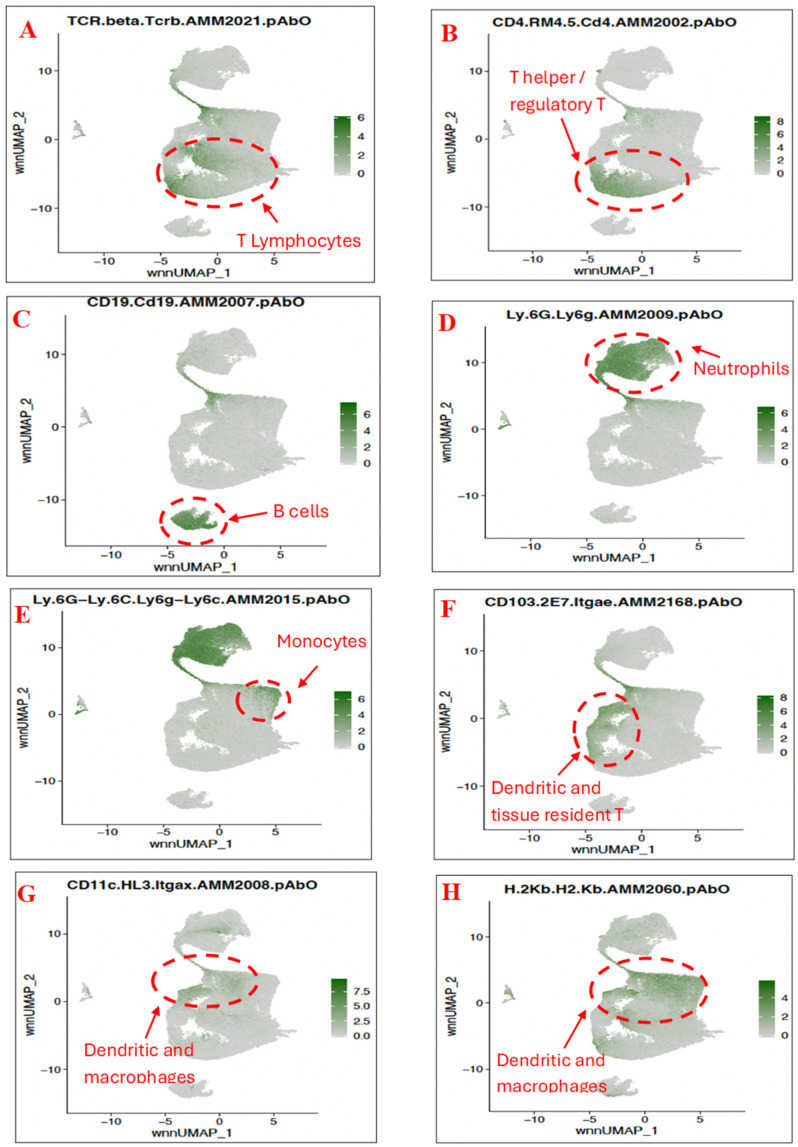

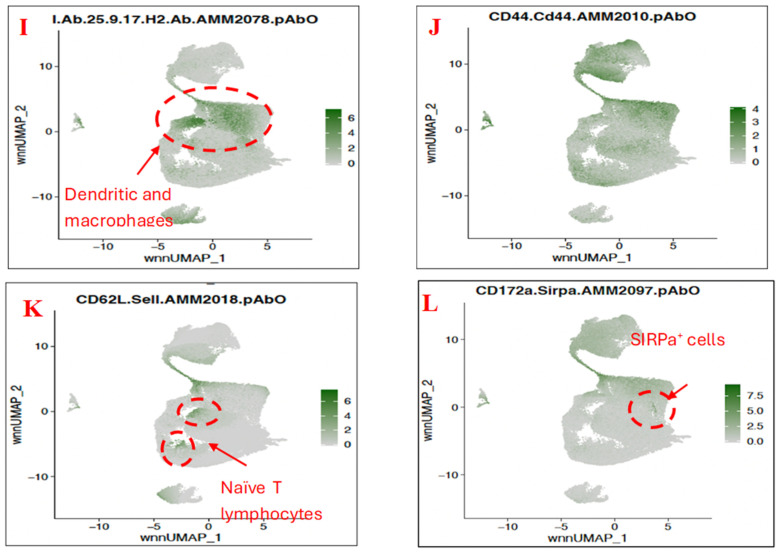
The localisation of the AbSeq antibodies, shown in Table A1, to the 2D expression profile of immune cells (UMAP) is shown in green in (**A**) for the TCR.beta AbSeq antibody showing T lymphocytes, in (**B**) for the CD4 AbSeq antibody showing Th and Treg, in (**C**) for the CD19 AbSeq antibody showing B cells, in (**D**) for the Ly6G AbSeq antibody showing neutrophils, in (**E**) for the Ly6G/Ly6C AbSeq antibody showing monocytes, in (**F**) for the CD103 AbSeq antibody showing DC and TRM T cells, in (**G**) for the CD11c AbSeq antibody showing DC and macrophages, in (**H**) for the H2kB AbSeq antibody showing DC and macrophages, in (**I**) for the I.Ab AbSeq antibody showing DC and macrophages, in (**J**) for the CD44 AbSeq antibody, in (**K**) for the SELL Abseq antibody showing possible naïve cell populations, and in (**L**) for the SIRPA Abseq antibody.

The two-dimensional transcriptomic profile of the CD45^+^ immune cell populations (leukocytes) found in the tumours of the four groups (sham controls, BH, reovirus, and combination) 24 h after treatment corresponded to 30 distinct clusters in a UMAP (Figure 3) which overlapped with the protein staining of the AbSeq antibodies as shown in Figure A2. The clusters were further annotated manually using biomarkers commonly associated with each immune cell type (as listed in Table 2A–D, Table A2 and Table A3). Neutrophils (seven clusters) (Table 2A,B), CD4^+^ lymphocytes (Th) (five clusters) (Table 2C), nine CD8^+^ lymphocytes clusters (Table 2D and Table A2) and two B cell clusters and single clusters of macrophages, inflammatory monocytes, SIRPα^+^ macrophages, natural killer (NK) cells, dendritic cells (DC), myeloid-derived suppressor cells (MDSC), and “leaky” damaged cells (Table A3) were identified.

This detailed analysis showed that some of these cell subsets were associated with an anti-immune pro-tumour gene expression phenotype. For example, all neutrophil subtypes (active, intermediate, inactive, transitioning neutrophils including subsets) overexpressed anti-immune genes associated with an N2-like phenotype, e.g., CXCR2, IL10R, CD177, OSM, TGF-βR (all but the active neutrophils subset cluster) and PDL1 (Table 2A). Cytotoxic T cell (Tcyt) clusters including the senescent Tcyt, terminally exhausted Tcyt, exhausted Tcyt, exhausted proliferating Tcyt, as well as the tissue-resident memory (TRM) CD8^+^T overexpressed one or more of the immune checkpoint genes LAG3, PDCD1, CTLA4, TIGIT, and TIM3 (Table 2D). A small but distinct set of macrophages showed significant gene downregulation (~76% of all its genes) and stained positive for the SIRPα protein (Table A3 and Figure A2L) suggesting the prior activation of the CD47-SIRPα signalling axis in this macrophage subset.

### 3.4. Relative Immune Cell Abundance Following Treatment

**Figure 4:**
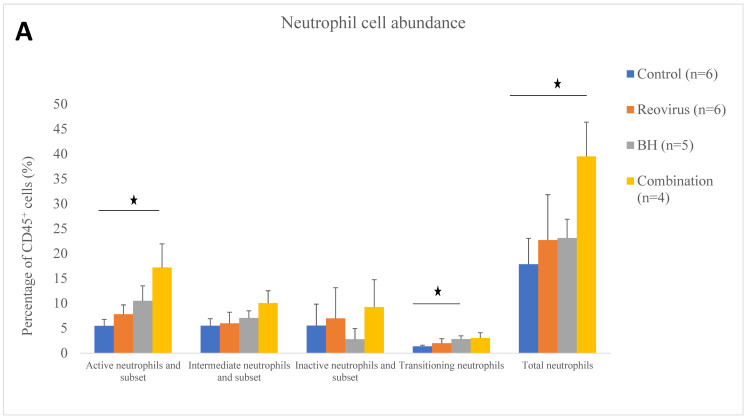

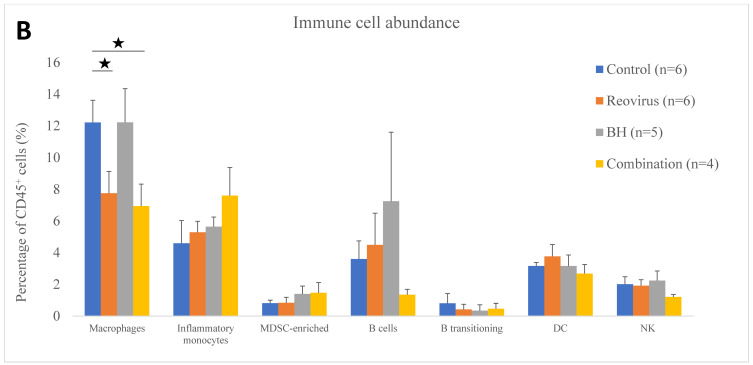

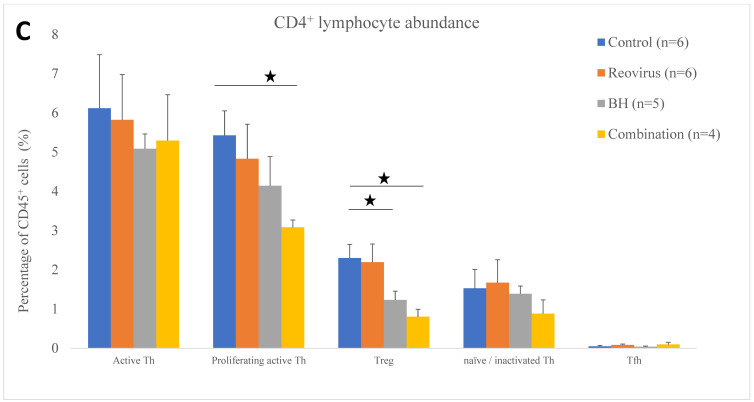

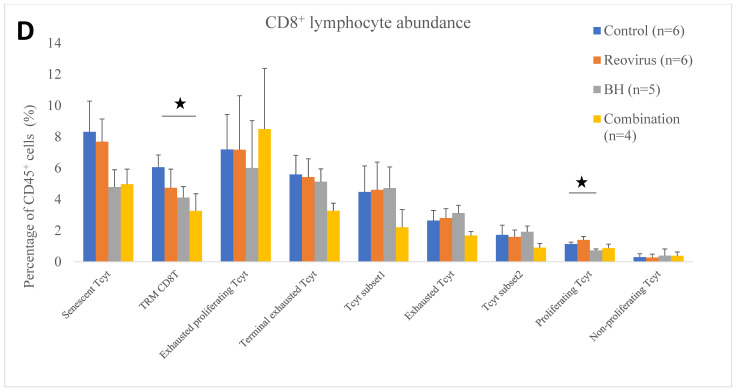
Relative abundance of immune cells in pancreatic tumours 24 h after treatment. The percentages of the various immune cells identified in this study are shown as a proportion of the total leukocytes (CD45^+^ cells) for (*n* = 6) sham-exposed control tumours (blue) and for (*n* = 6) subjects in the reovirus (orange), (*n* = 5) BH (grey), and (*n* = 4) combined treatment (yellow) groups in (**A**) for neutrophils, in (**B**) for macrophages, inflammatory monocytes, MDSC-enriched cells, B cells, B transitioning cells, DC and NK cells, in (**C**) for CD4^+^ lymphocytes, and in (**D**) for CD8^+^ lymphocytes. Results are shown as averages ± SEM. Statistical significance (*p* < 0.05) is denoted with an asterisk.

**Figure A3:**
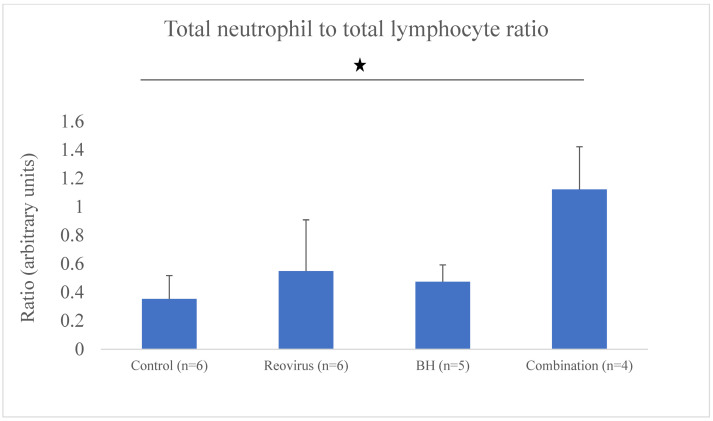
The total neutrophil to total lymphocyte ratio in the pancreatic tumours 24 h after sham exposures and treatments with reovirus, BH, and the combined BH and reovirus. Results are shown as averages ± SEM. Statistical significance is denoted with an asterisk and is assumed at *p* < 0.05.

**Figure A4:**
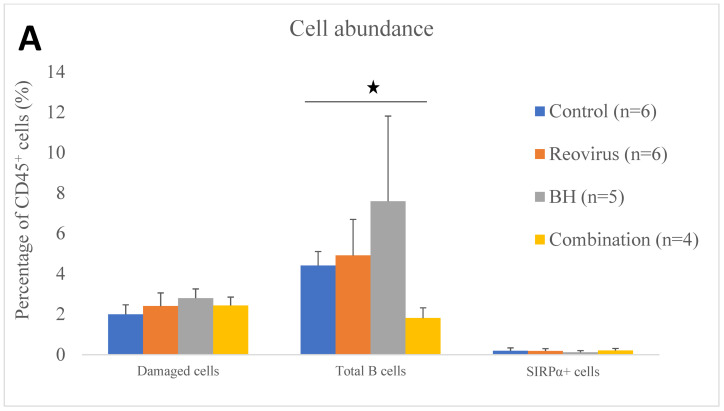

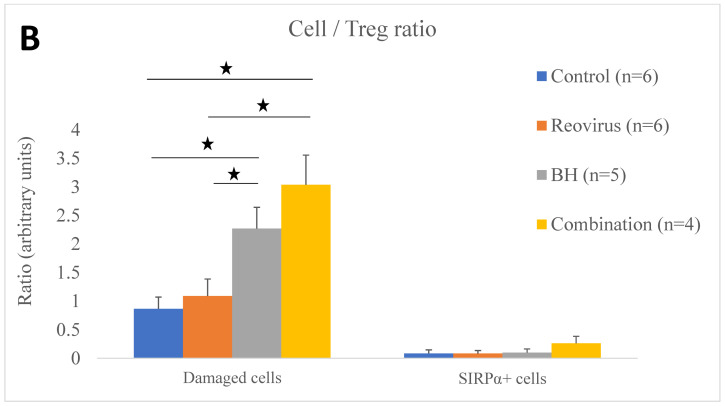
The percentages of cells with leaky membranes (damaged), SIRPα^+^ cells, and total B cells as a proportion of the total leukocytes (CD45^+^ cells) (**A**), and the ratios of the damaged and SIRPα^+^ cells to Treg (**B**) in the pancreatic tumours 24 h after treatments are shown for sham-exposed control tumours (blue) and for (*n* = 6) subjects in the reovirus (orange), (*n* = 5) BH (grey), and (*n* = 4) combined treatment (yellow) groups. Results are shown as averages ± SEM. Statistical significance is denoted with an asterisk and is assumed at *p* < 0.05.

The relative abundance of the 30 immune cell subtypes (compared to the total leukocyte abundance) in the tumours 24 h after sham, BH, reovirus, and combination treatments is shown in Figure 4, Figure A3 and Figure A4A and Table 3. The statistical significance of the results for each cell type in the treatment groups compared to the sham-exposed subjects is shown in Table A4. Statistically significant increases were seen in the abundance of the transitioning neutrophils (2.1-fold) for the BH group compared to that in the control group (Figure 4A), as well as for the aggregated active neutrophils (3.1-fold) (Figure 4A), total neutrophils (active, intermediate, inactive, and transitioning clusters and subsets) (2.2-fold) (Figure 4A), and the total neutrophil to total lymphocyte (CD8 T cells, CD4 T cells, NK, and B cells) ratio (NLR) (3.2-fold) (Figure A3) in the tumours of the combination group compared to the sham-exposed subjects. When the reovirus group and the BH group were compared to the sham-exposed group, no statistically significant differences were seen in the neutrophil abundance or the NLR.

Statistically significant decreases in the abundance of macrophages (1.8-fold) (Figure 4B), proliferating active Th (1.8-fold) (Figure 4C), regulatory T cells (Treg) (2.9-fold) (Figure 4C), TRM CD8T (1.9-fold) (Figure 4D), and total B cells (B cells + B transitioning) (2.4-fold) (Figure A4A) were seen in the combination-treated subjects compared to the sham-exposed ones. Statistically significant decreases were also seen for the Treg (1.9-fold) (Figure 4C) and proliferating Tcyt (1.6-fold) (Figure 4D) of the BH-exposed group and for the macrophages (1.6-fold) of the reovirus treated group (Figure 4B) compared with the sham-exposed group.

**Figure 5:**
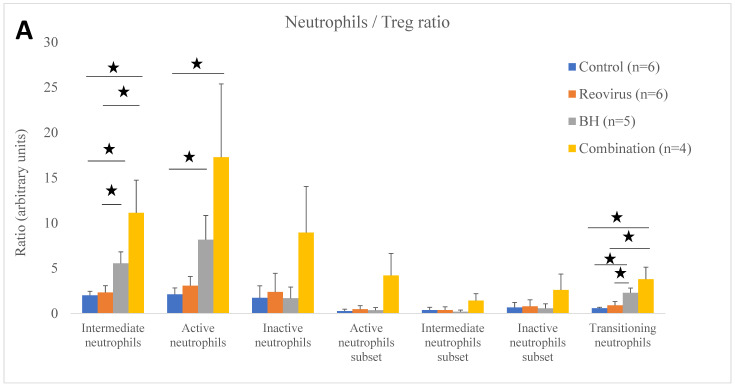

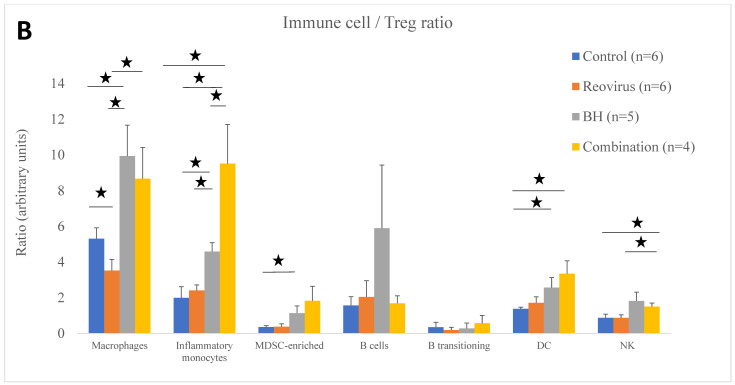

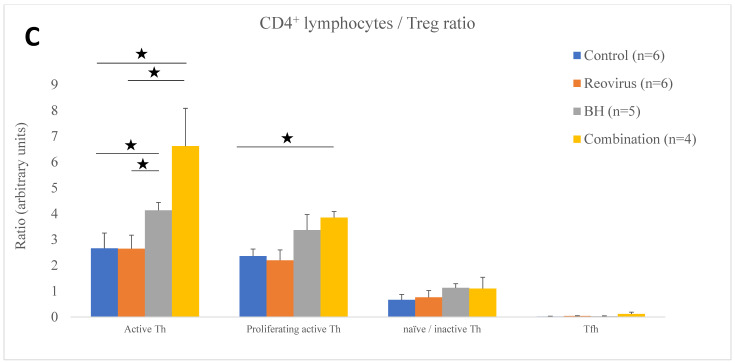

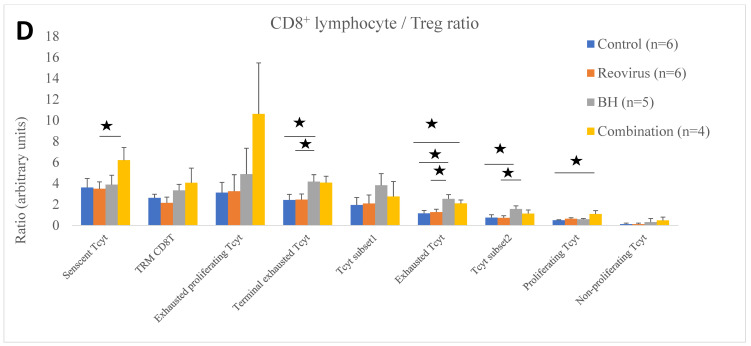
The immune cells/Treg ratios for pancreatic tumours 24 h after treatment. The ratios of the various immune cell types to Treg are shown in (**A**) for neutrophils, in (**B**) for macrophages, inflammatory monocytes, MDSC-enriched cells, B cells, B transitioning cells, DC and NK cells, in (**C**) for CD4^+^ lymphocytes, and in (**D**) for CD8^+^ lymphocytes. Results are shown as averages ± SEM for each cell type in sham-exposed tumours (*n* = 6) (blue) and in KPC tumours exposed to reovirus (*n* = 6) (orange), histotripsy (*n* = 5) (grey), and combination treatments (*n* = 4) (yellow). Statistical significance is denoted with an asterisk and is assumed at *p* < 0.05.

Ultrasound exposures (BH and combination treatments) increased the balance of immune cells to Treg cells compared to the sham-exposed group (Figure 5 and Figure A4B and Table 4 and Table A5). For the BH and combination groups compared to the sham-exposed controls, statistically significant increases were seen for the intermediate neutrophils (2.8- and 5.5-fold, respectively) (Figure 5A), transitioning neutrophils (3.9- and 6.4-fold) (Figure 5A), inflammatory monocytes (2.3- and 4.8-fold) (Figure 5B), DC (1.9- and 2.4-fold) (Figure 5B), active Th (1.6- and 2.5-fold) (Figure 5C), and exhausted Tcyt (2.2- and 1.8-fold) (Figure 5D). In addition, for the combination vs. sham-exposed groups only increases were seen for the ratios of the NK cells (1.7-fold) (Figure 5B), proliferating active Th (1.6-fold) (Figure 5C), and proliferating Tcyt (2.2-fold) (Figure 5D) to Treg. For the BH-exposed tumours, increases were seen for the ratios of the macrophages (1.9-fold) (Figure 5B), MDSC-enriched cells (3.2-fold) (Figure 5B), terminal exhausted Tcyt (1.7-fold) (Figure 5D), and Tcyt subset2 (2.1-fold) (Figure 5D) over Treg. In the reovirus group, a decrease in the macrophages/Treg (1.5-fold) (Figure 5B) was seen compared to the sham-exposed subjects.

Statistically significant differences in the ratio of immune cells to Treg were also seen between treatment groups. Compared to the reovirus-treated subjects, the combined treatments increased the ratios of the intermediate neutrophils to Treg (4.8-fold), and transitioning neutrophils (4.2-fold) (Figure 5A), inflammatory monocytes (4.0-fold), macrophages (2.5-fold), NK cells (1.7-fold) (Figure 5B), active Th (2.5-fold) (Figure 5C), and senescent Tcyt (1.8-fold) (Figure 5D). Also, the combined treatments increased the ratio of the inflammatory monocytes to Treg (2.1-fold) when compared to the BH-only exposures (Figure 5B). The BH exposures, compared to the reovirus-only treatments, resulted in increased ratios of intermediate neutrophils to Treg (2.4-fold), and transitioning neutrophils (2.5-fold) (Figure 5A), macrophages (2.8-fold), inflammatory monocytes (1.9-fold) (Figure 5B), active Th (1.6-fold) (Figure 5C), terminal exhausted Tcyt (1.7-fold), exhausted Tcyt (2.0-fold), and Tcyt subset2 (2.2-fold) (Figure 5D).

### 3.5. Effects of Treatments on Differential Gene Expression in All Immune Cell Clusters

Differential gene expression (DEG) in all clusters treated with reovirus, BH, BH + reovirus, and in sham-exposed controls 24 h after treatment was investigated to characterise in detail the transcriptomic profile of the tumoural immune cell infiltrates.

Compared to the sham-exposed controls, reovirus treatments differentially modulated 655 genes in all clusters (Table 5), causing significant DEG in macrophages and inflammatory monocytes (>200 genes differentially modulated in each cell type), active Th and proliferating active Th (≥35 genes differentially modulated in each cell type), exhausted proliferating Tcyt (18 genes upregulated), and active neutrophils (37 genes upregulated). BH treatments alone modulated the expression of 608 genes (Table 5), particularly in active and intermediate neutrophils (>150 genes upregulated in each cell type) and in the exhausted proliferating Tcyt (30 genes upregulated). Unlike the reovirus treatments, BH caused no significant changes in the gene expression of inflammatory monocytes and downregulated 105 macrophage and 38 DC genes compared to control subjects (Table 5).

**Figure 6:**
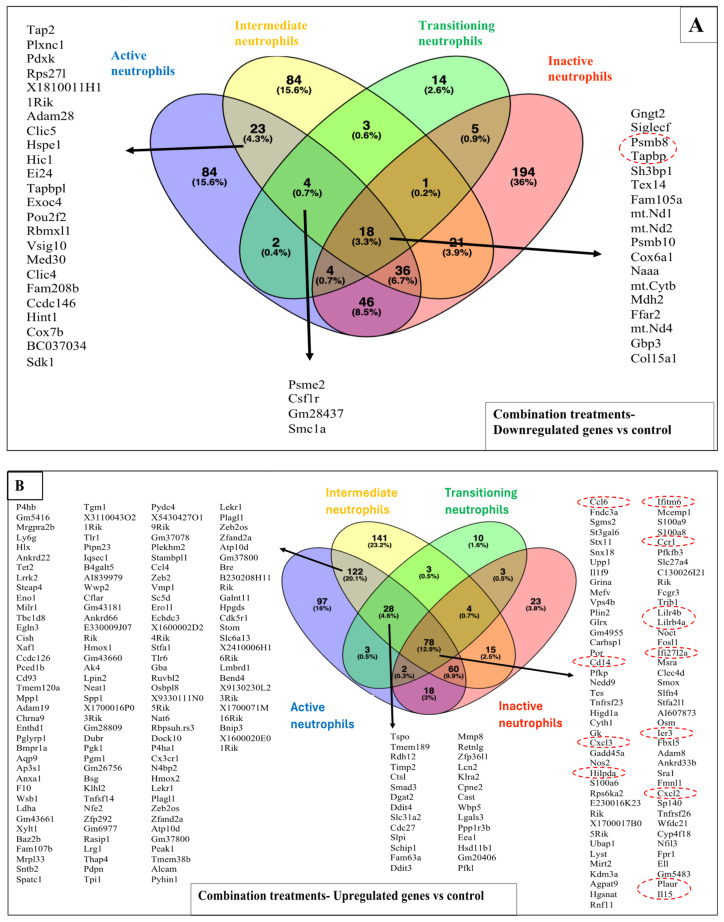

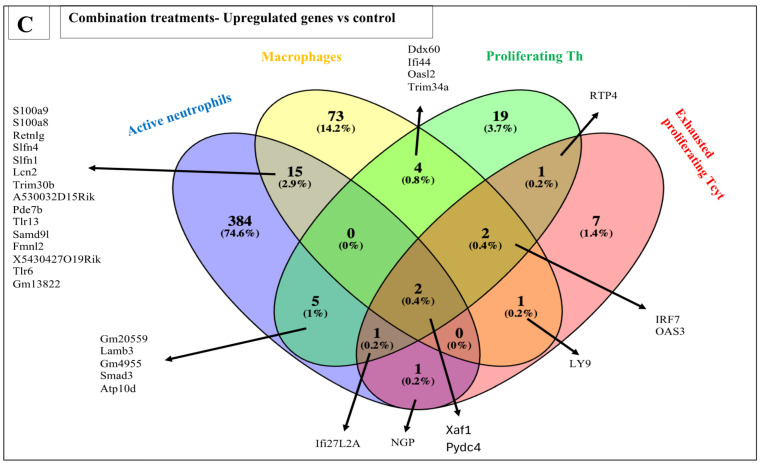
Commonly regulated genes in immune cell clusters 24 h after treatment. PDAC KPC tumours were sham-exposed (*n* = 6) or treated with the combined reovirus + BH (*n* = 4) and excised 24 h later. Combination vs. control DEG results from these tumours were analysed with Venn diagrams (**A**–**C**) to illustrate commonly regulated genes among different cell clusters in the BH + reovirus-treated tumours. Each coloured ellipse in the Venn diagram indicates the total number of modulated genes within the indicated cluster, and the subset that overlaps between the ellipses represents the number of commonly modulated genes among the clusters. These results are shown in (**A**) for commonly downregulated genes in active and intermediate neutrophils (23 genes), in active, intermediate, and transitioning neutrophils (4 genes), and in all neutrophil clusters (18 genes); in (**B**) for commonly upregulated genes in active and intermediate neutrophils (122 genes), in active, intermediate and transitioning neutrophils (28 genes), and in all neutrophil clusters (78 genes); and in (**C**) for commonly upregulated genes in macrophages, active neutrophils, proliferating active Th, and exhausted proliferating Tcyt (2 genes), as well as in various other combinations of these 4 cell types thereafter. Genes were assumed significantly regulated only if their AvgLog_2_FC expression > 1 and their *p*-adjusted value < 0.05 in the combination vs. control groups. Genes referred to in the Discussion are highlighted in red to ease their identification here.

Compared to the sham-exposed controls, the combined treatments differentially modulated more genes (>2-fold) than the reovirus-alone and BH-alone treatments combined. In total, the transcription of ~2600 genes was affected among all cell clusters (Table 5) with ~1700 genes significantly increasing their expression and ~950 genes decreasing their expression after the combined treatments. The cell types with the highest DEG were the active, intermediate, and inactive neutrophils (>500 genes differentially modulated in each cell type), the transitioning neutrophils and cells of monocytic origin such as the macrophages and inflammatory monocytes (>140 genes differentially modulated), followed by the Th cells and various Tcyt clusters (Table 5). Also, after the combined treatments, 18 genes were commonly downregulated (Figure 6A), and 78 genes were commonly upregulated (Figure 6B) among the active, intermediate, transitioning, and inactive neutrophils compared to the controls. In addition, the XAF1 and PYDC4 genes were commonly upregulated among the active neutrophils, macrophages, proliferating Th, and exhausted proliferating Tcyt after the combination treatments (Figure 6C).

**Figure A5:**
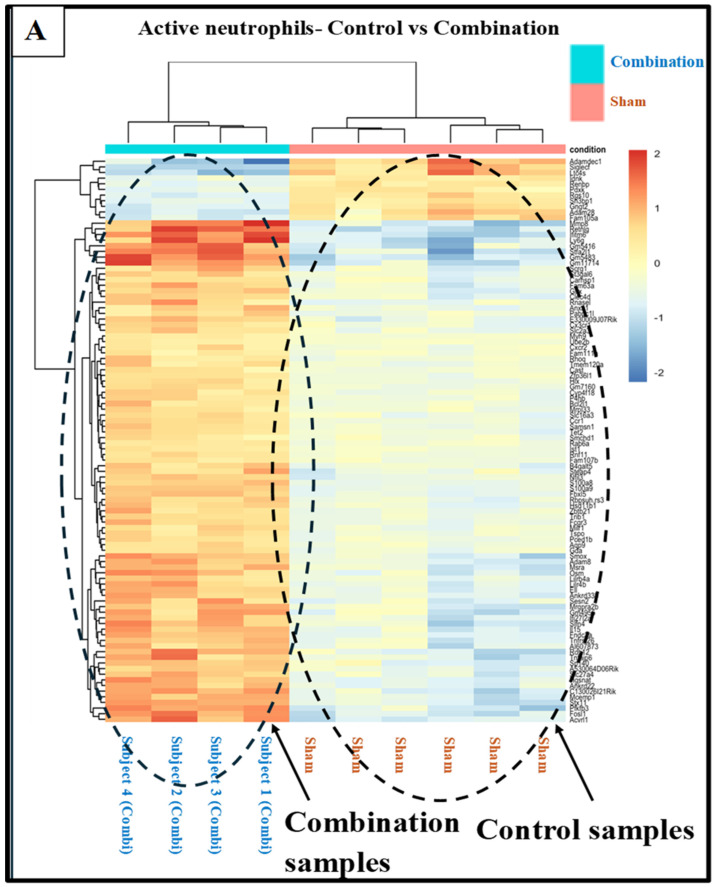

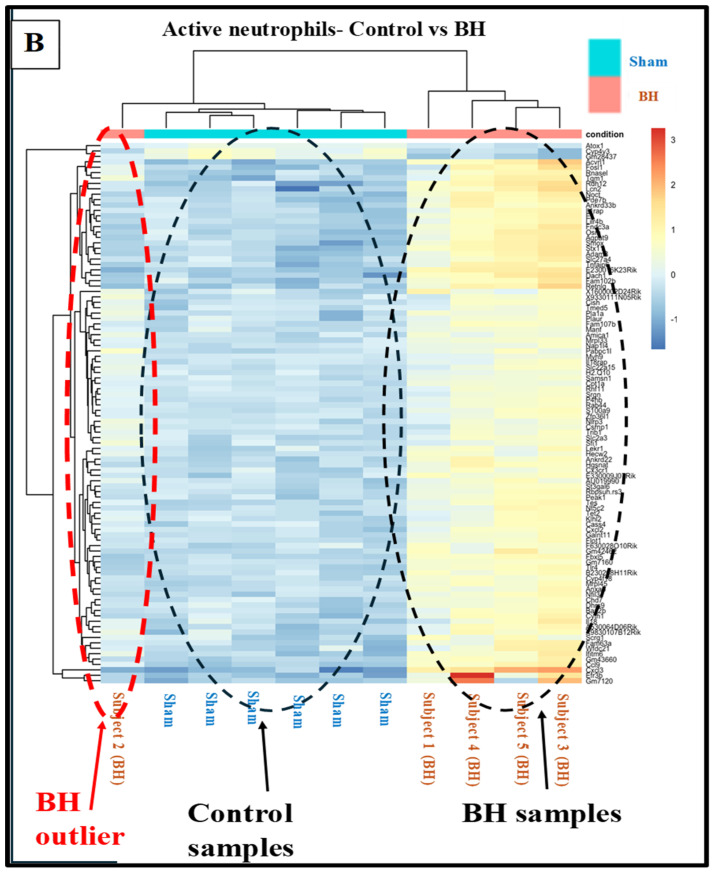

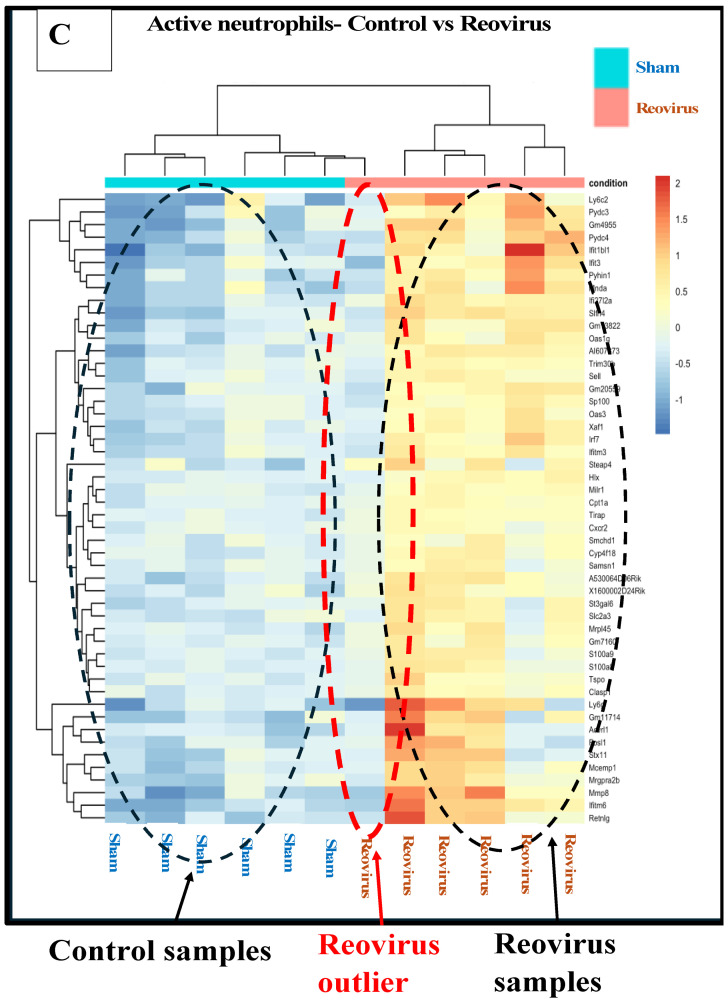
Heatmaps of the top 100 differentially expressed genes in active neutrophils for every subject are shown in (**A**) for the control vs. combination groups, in (**B**) for the control vs. BH groups, and in (**C**) for the control vs. reovirus groups.

Heat maps of the top DEG for each subject showed reasonable gene expression homogeneity among subjects within each treatment and control group. For example, for active neutrophils in the sham vs. combination groups, the top DEG in all sham-exposed subjects were grouped, and similarly, the top DEG in all combination-treated subjects were also grouped (Figure A5A). In the control vs. BH active neutrophils heat map, one outlier of the five BH-treated subjects (subject 2 (Table 1)) shared DEG similarities with the controls (Figure A5B), and the same was true for one outlier from the six reovirus-treated subjects in the control vs. reovirus active neutrophils heat maps (Figure A5C).

### 3.6. Functional Enrichment Analysis in KPC Tumours

#### 3.6.1. Reovirus Treatments vs. Sham-Exposed Controls

**Figure A6:**
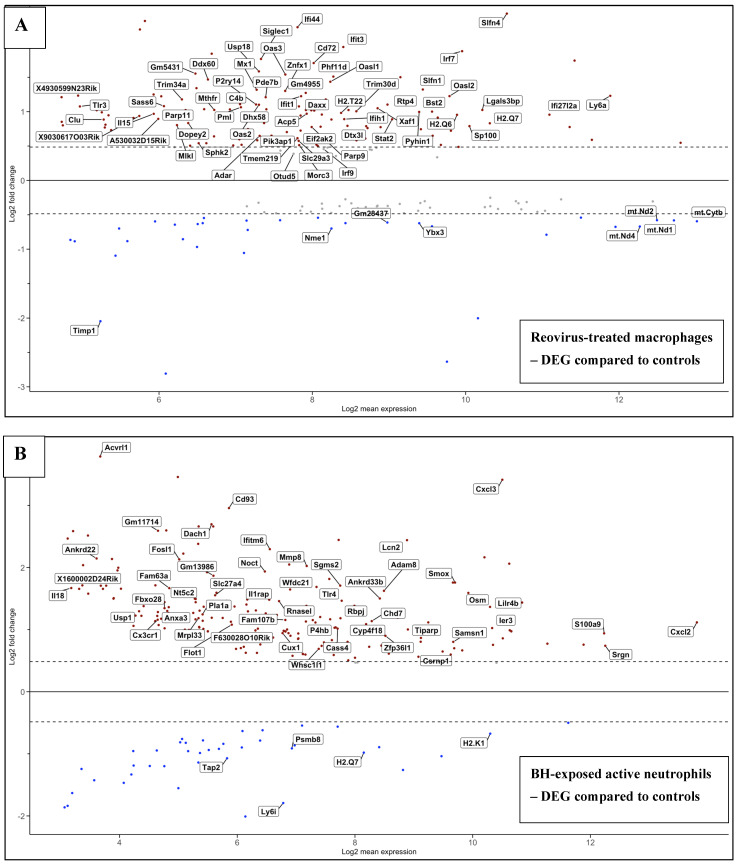

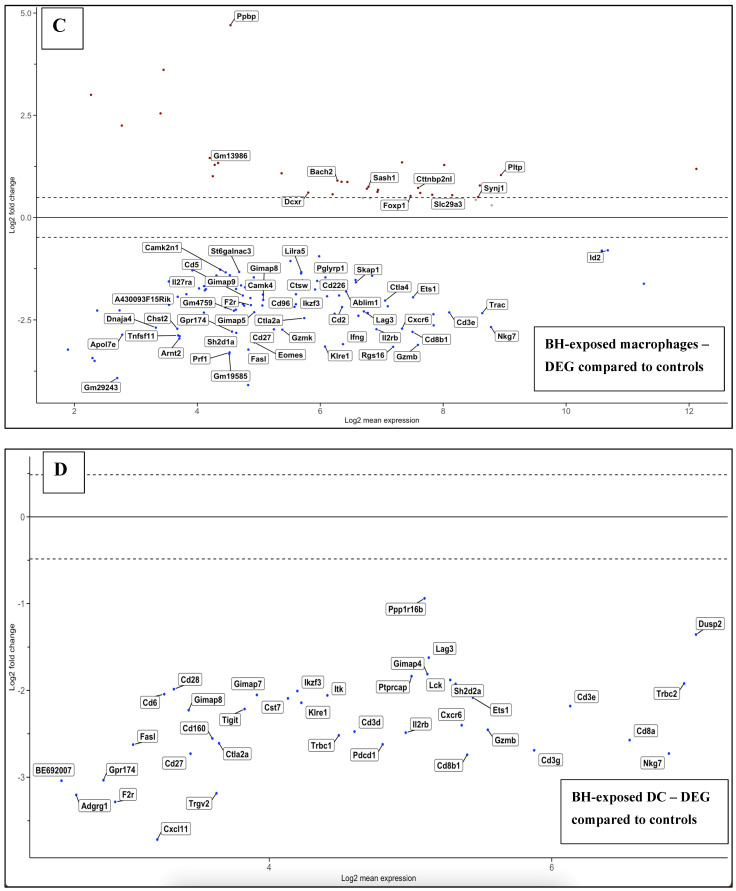

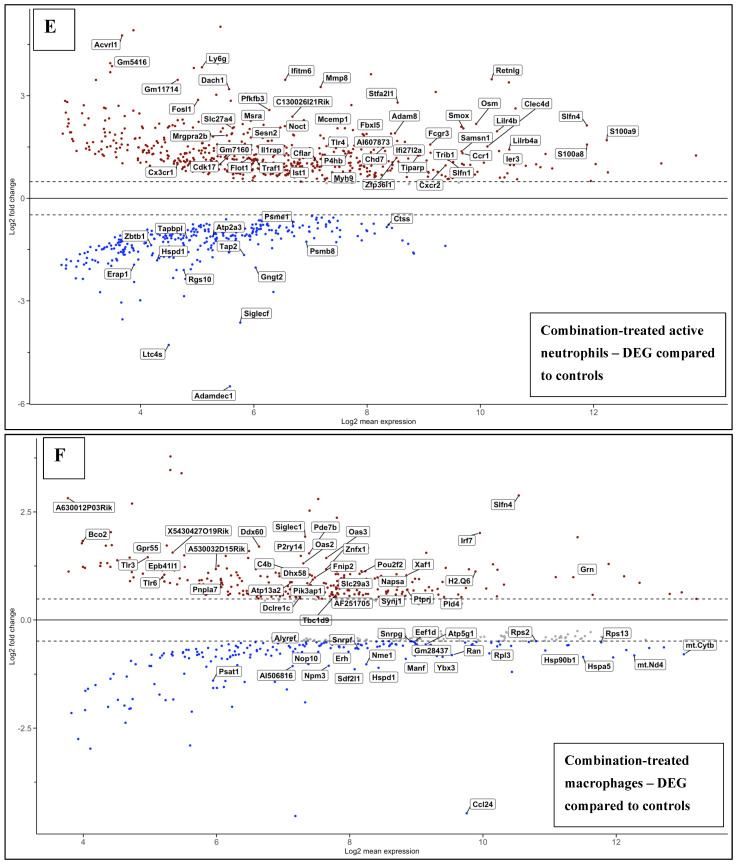
DEG analysis in KPC tumours 24 h after sham exposure (*n* = 6) or treatments with reovirus (*n* = 6), BH (*n* = 5), or combined BH and reovirus (*n* = 4). DEG results are shown for the reovirus vs. sham-exposed macrophages in (**A**), BH vs. sham-exposed active neutrophils in (**B**), BH vs. sham-exposed macrophages in (**C**), BH vs. sham-exposed DC in (**D**), combination vs. sham-exposed active neutrophils in (**E**), and combination vs. sham-exposed macrophages in (**F**). Results shown are ggMAP plots showing the top 50 hits along with the selected genes mentioned in the “Functional enrichment analysis—All treatments” section of the discussion, *p*-adjusted < 0.05.

**Figure A7:**
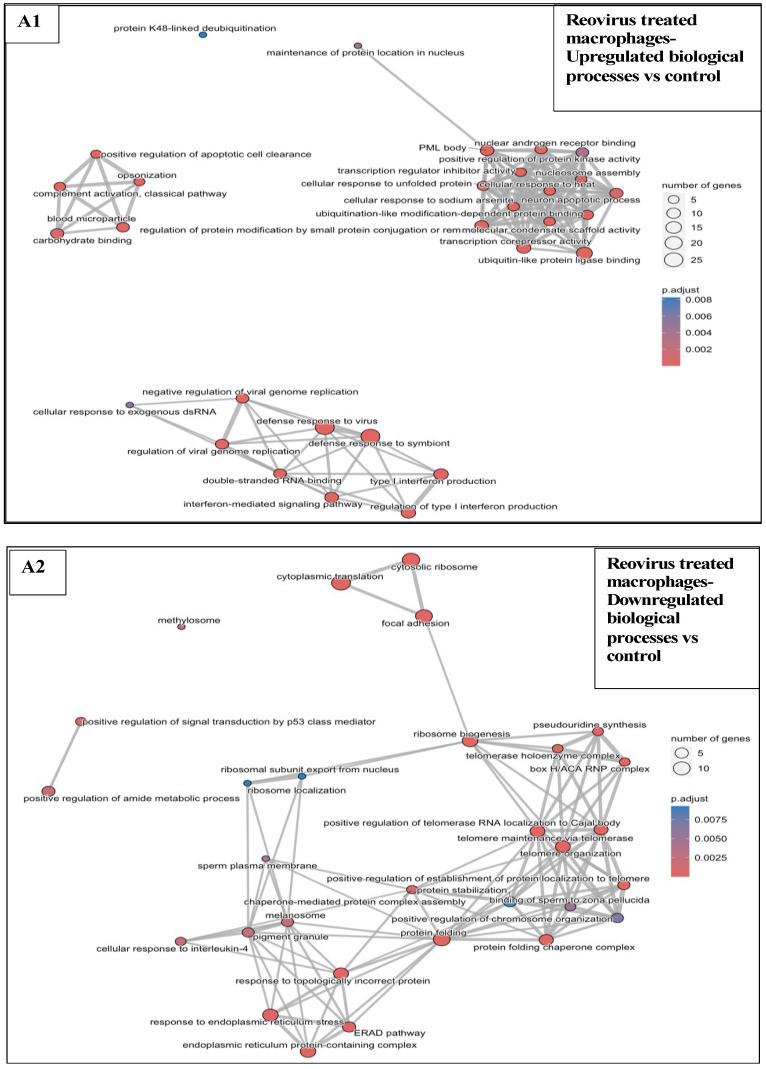

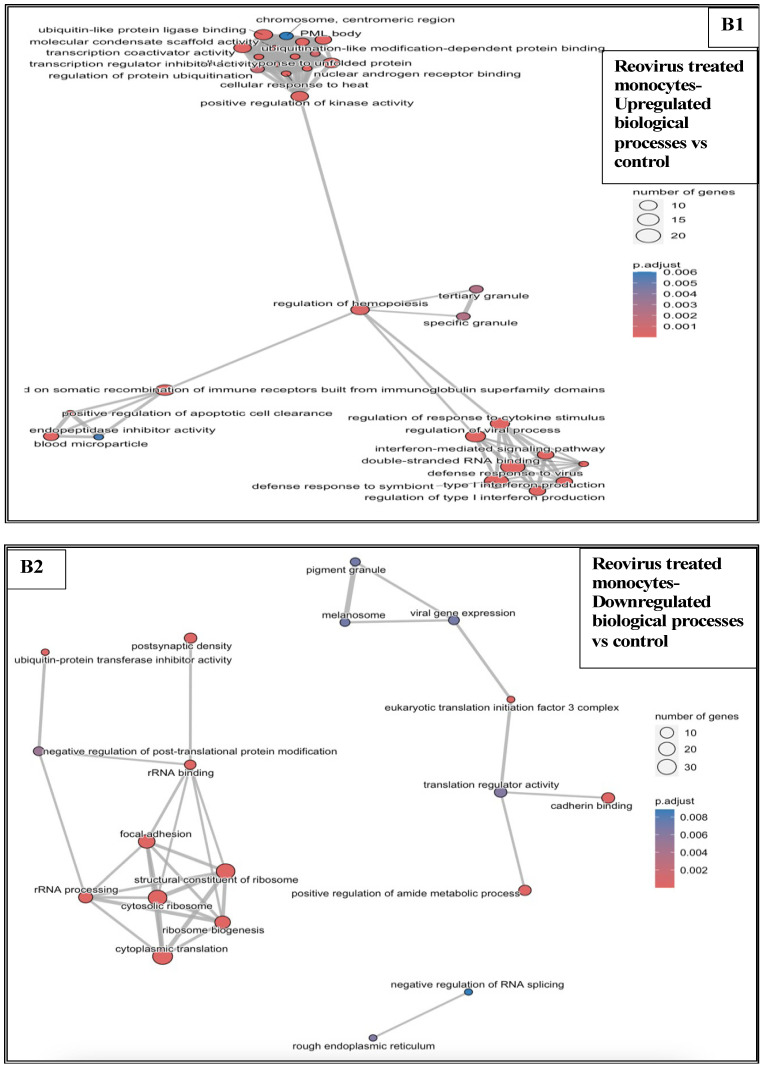

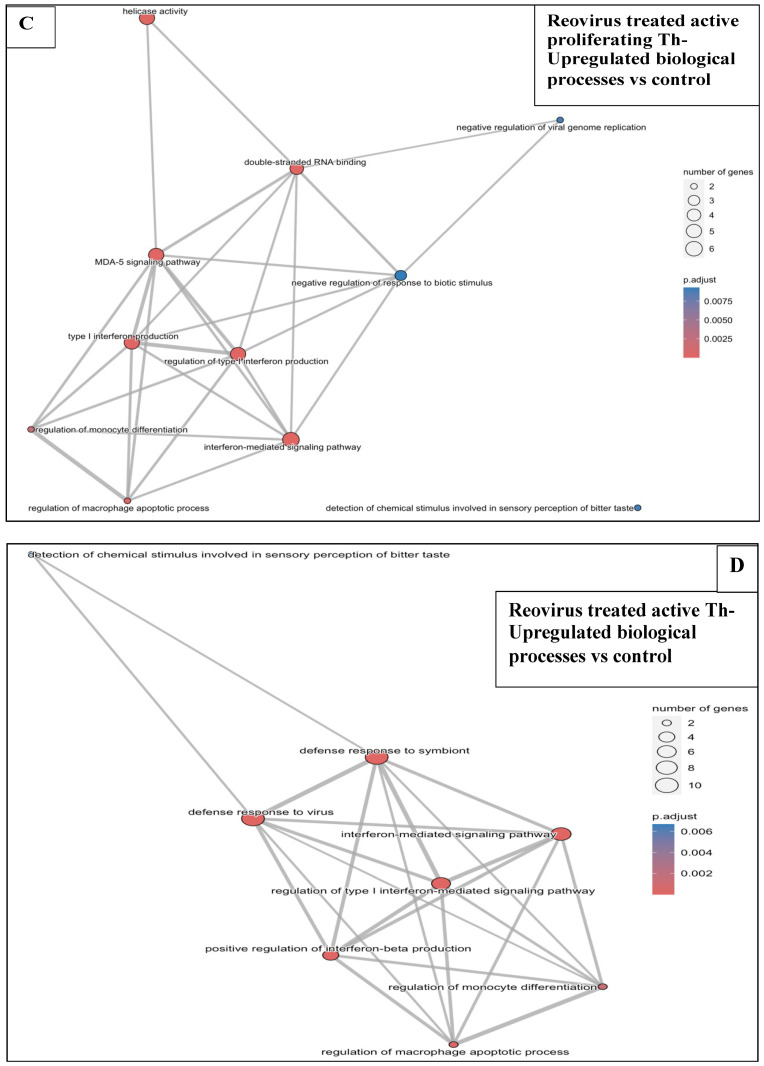

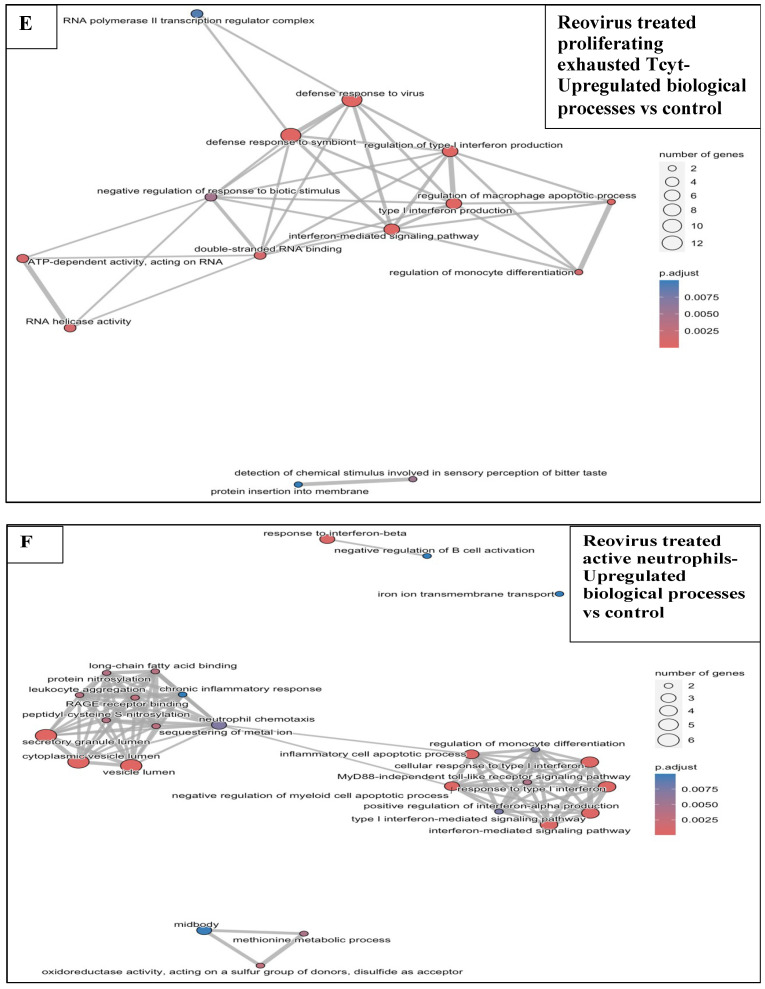
Functional enrichment analysis of DEG in KPC tumours 24 h after treatment with reovirus. KPC tumours were sham-exposed (*n* = 6) or treated with reovirus (*n* = 6) and excised 24 h later. DEG results from reovirus vs. control tumours were functionally analysed to identify the biological processes that are associated with DEG. These are shown for reovirus-treated subjects compared to sham-exposed subjects in (**A1**) for upregulated genes and (**A2**) downregulated genes in macrophages, in (**B1**) for upregulated genes and (**B2**) for downregulated genes in inflammatory monocytes, in (**C**) for active proliferating active Th, in (**D**) for active Th, in (**E**) for proliferating exhausted Tcyt, and in (**F**) for active neutrophils. Results are shown for DEG with *p*-adjusted values < 0.05 between the reovirus and sham-exposed tumours and are presented as functional enrichment plots of these DEG. The number of Ensembl gene IDs is represented by the open circle size, and the colour-coded *p*-adjusted values show the significance range.

In macrophages, inflammatory monocytes, active Th, proliferating active Th, exhausted proliferating Tcyt, and active neutrophils, 24 h after treatment of the KPC tumours with reovirus, overexpressed genes (an example shown for macrophages in Figure A6A) were associated with the innate immune response including type I interferon (IFN) signalling and production, and defence responses to viruses (Figure A7A–F). In addition, overexpressed genes were associated with the complement activation and apoptotic cell clearance in macrophages (Figure A7A), with the regulation of the MDA-5 signalling in active proliferating Th (Figure A7C), and with cytoplasmic vesicles, the receptor for advanced glycation end-products (RAGE) binding and responses to IFN-β in active neutrophils (Figure A7F).

#### 3.6.2. BH Exposures vs. Sham-Exposed Controls

**Figure 7:**
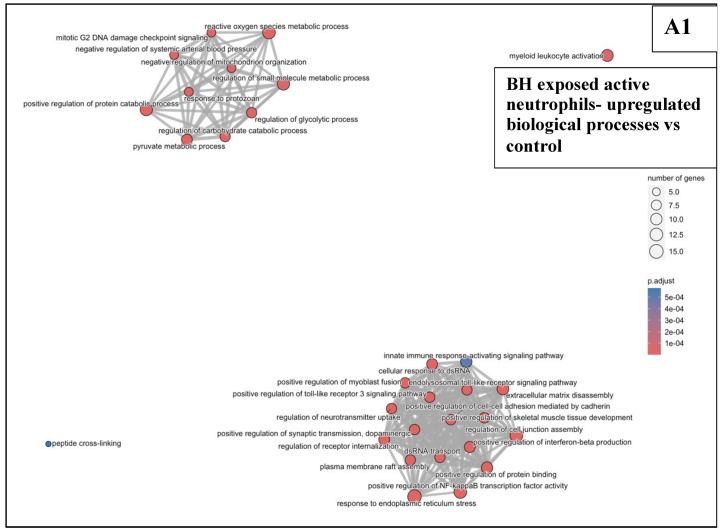

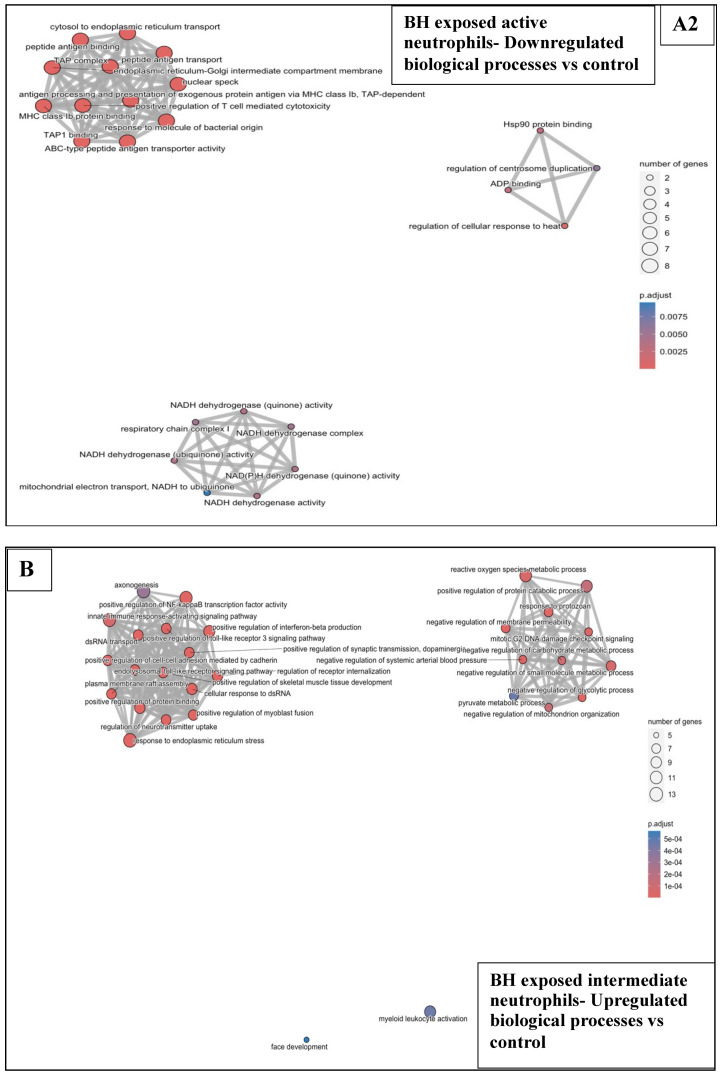

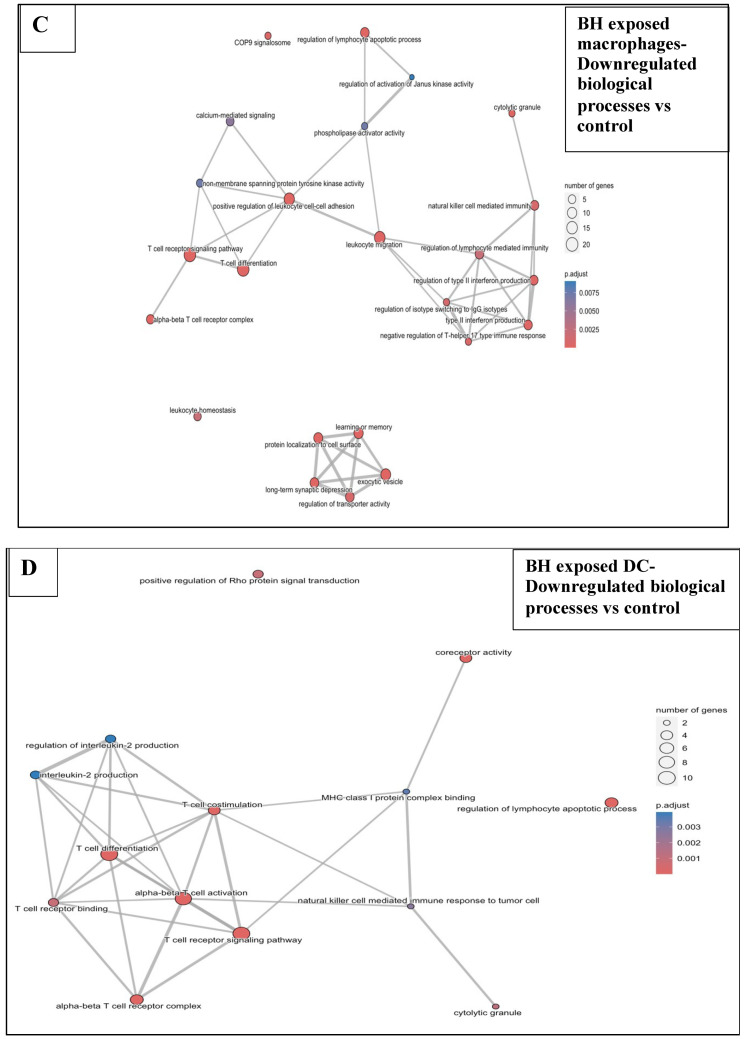
Functional enrichment analysis of DEG in KPC tumours 24 h after BH treatment. KPC tumours were sham-exposed (*n* = 6) or treated with BH (*n* = 5) and excised 24 h later. DEG results from BH vs. control tumours were functionally analysed to identify some of the biological processes that are associated with DEG. These are shown for BH vs. sham-exposed subjects in (**A1**) for upregulated genes and (**A2**) for downregulated genes in active neutrophils, in (**B**) for intermediate neutrophils, in (**C**) for macrophages, and in (**D**) for DC. Results are shown for DEG with *p*-adjusted values < 0.05 between the BH and sham-exposed tumours and are presented as functional enrichment plots of these DEG. The number of Ensembl gene IDs is represented by the open circle size, and the colour-coded *p*-adjusted values show the significance range.

BH treatments caused the overexpression of genes (an example shown for active neutrophils in Figure A6B) associated with the innate immune response, for example, TLR3 signalling, NF-kB signalling, and IFN-β production, in active (Figure 7A1) and intermediate (Figure 7B) neutrophils 24 h after treatment. But here at least some downregulated genes (Figure A6B) were associated with the transporter associated with antigen processing (TAP)-dependent class I major histocompatibility (MHC) complex, the ABC-type peptide antigen transported activity, and T cell-mediated cytotoxicity (Figure 7A2). In macrophages, downregulated genes (Figure A6C) were associated with type II IFN production, NK cell and lymphocyte-mediated immunity, the α/β T cell receptor complex, T cell receptor signalling, and T cell differentiation, migration, and cell adhesion (Figure 7C). In DC, BH treatments downregulated genes (Figure A6D) that were associated with the α/β T cell activation, IL-2 production, T cell costimulation differentiation activation and signalling, and a small number of genes associated with the TAP and class I MHC complexes, and the NK mediated anti-cancer immune response (Figure 7D).

#### 3.6.3. Combination Treatments vs. Sham-Exposed Controls

**Figure 8:**
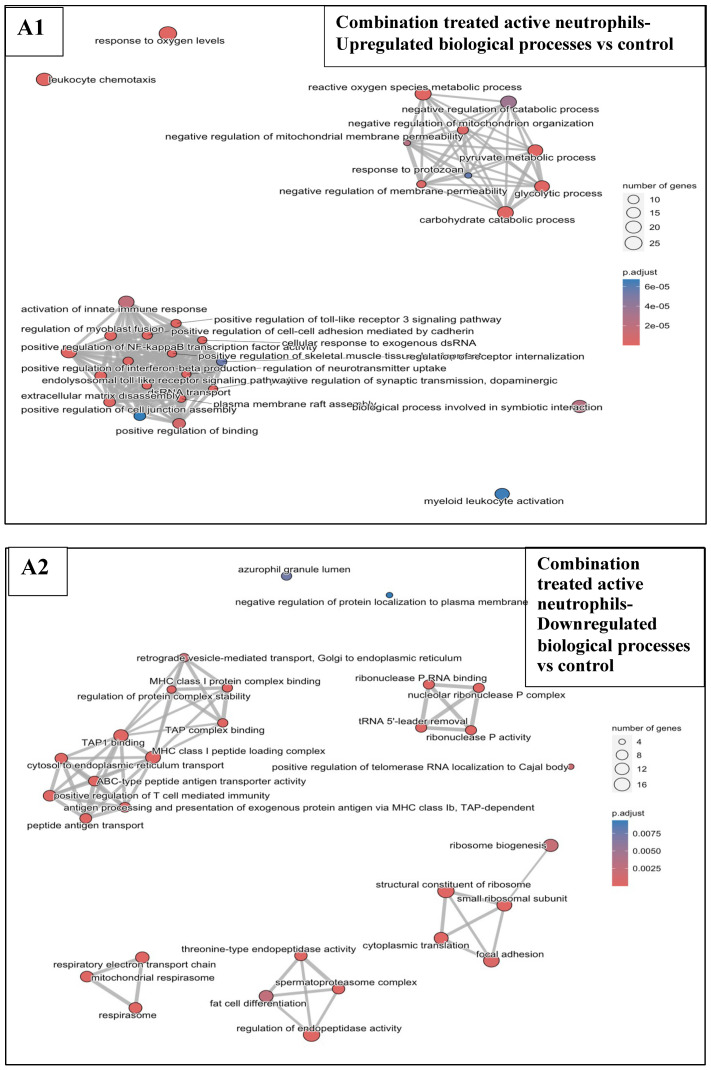

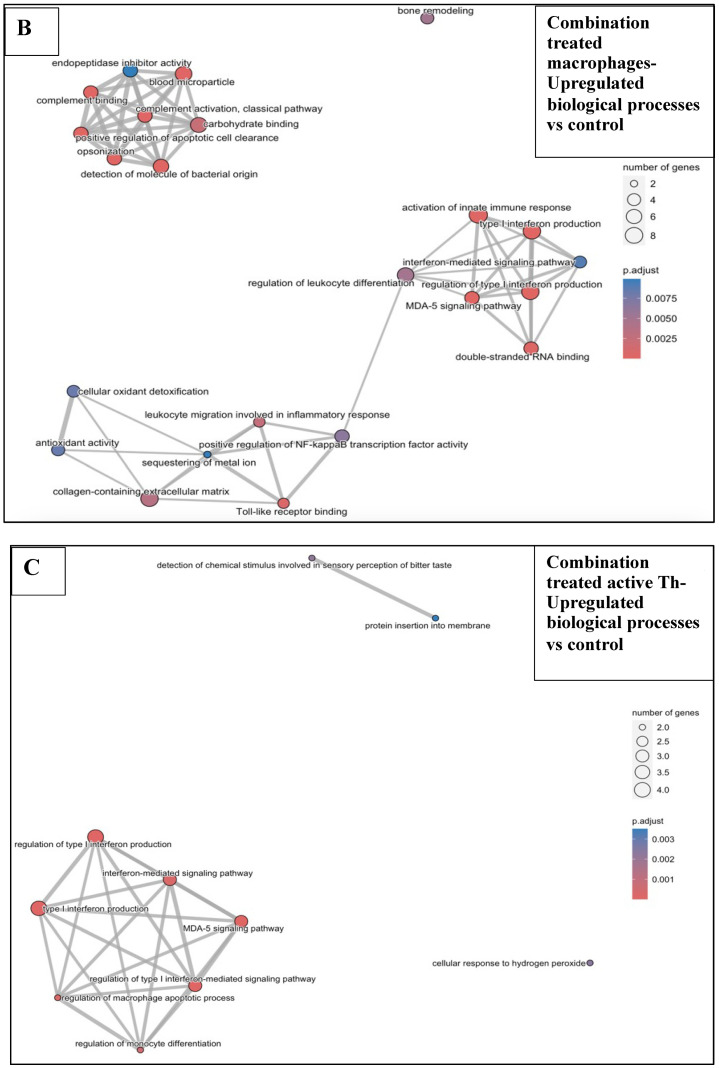

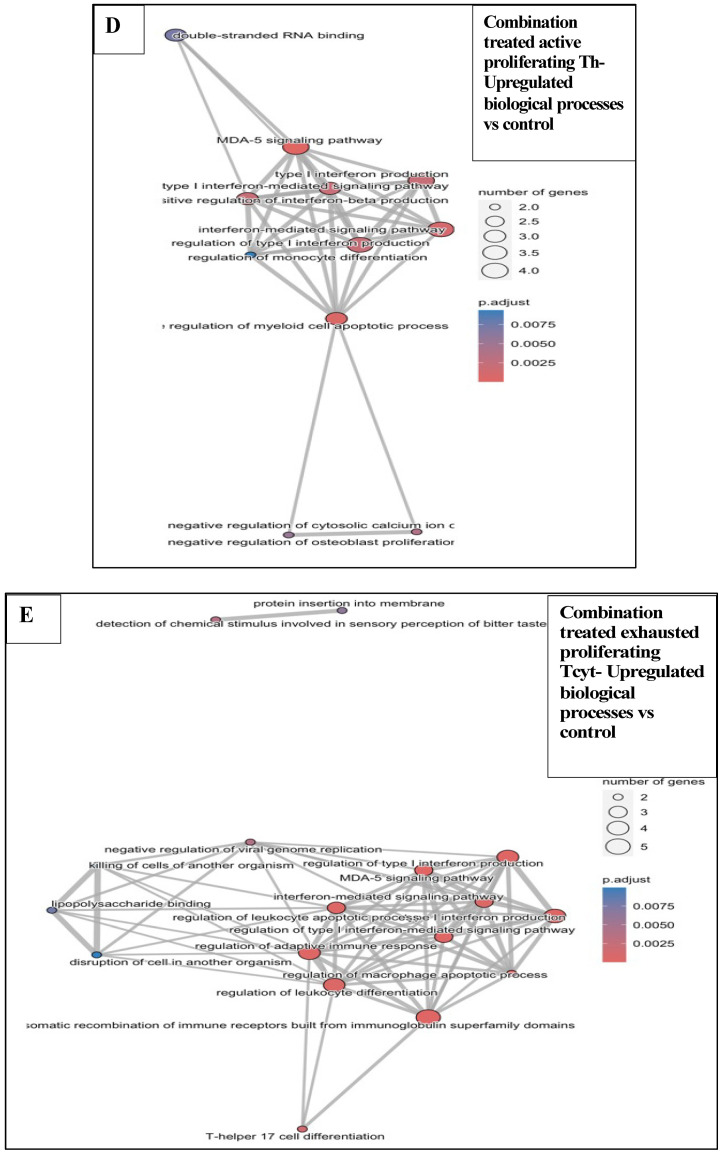

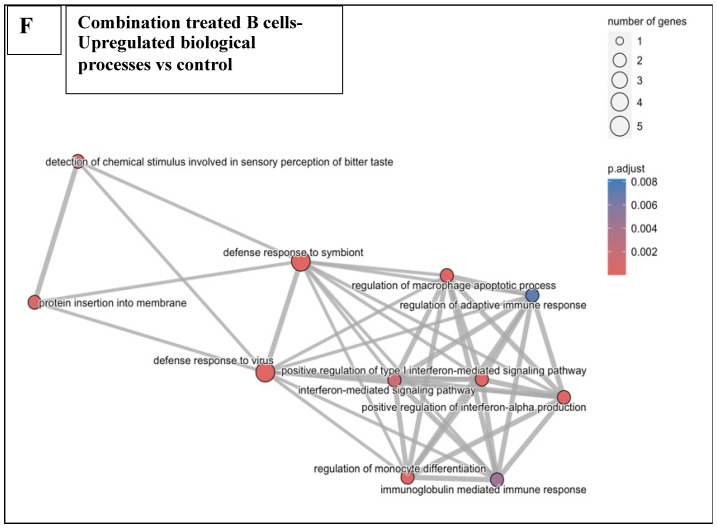
Functional enrichment analysis of DEG in KPC tumours 24 h after treatment with the combined BH + reovirus. KPC tumours were sham-exposed (*n* = 6) or treated with BH + reovirus (*n* = 4) and excised 24 h later. DEG results from the combined vs. control tumours were functionally analysed to identify biological processes that are associated with DEG. These are shown for the combined-treated subjects compared to the sham-exposed subject in (**A1**) for upregulated genes and (**A2**) for downregulated genes in active neutrophils, in (**B**) for macrophages, in (**C**) for active Th, in (**D**) for proliferating active Th, in (**E**) for exhausted proliferating Tcyt, and in (**F**) for B cells. Results are shown for DEG with *p*-adjusted values < 0.05 between the combined and sham-exposed tumours and are presented as functional enrichment plots of these DEG. The number of Ensembl gene IDs is represented by the open circle size, and the colour-coded *p*-adjusted values show the significance range.

**Figure A8:**
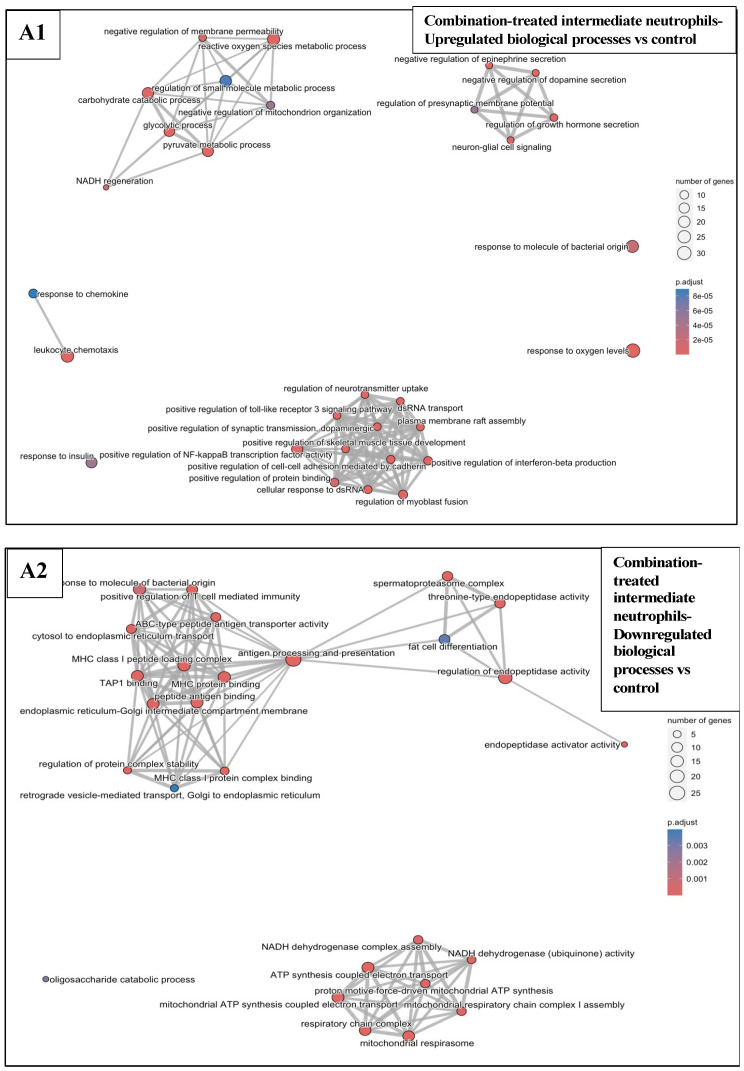

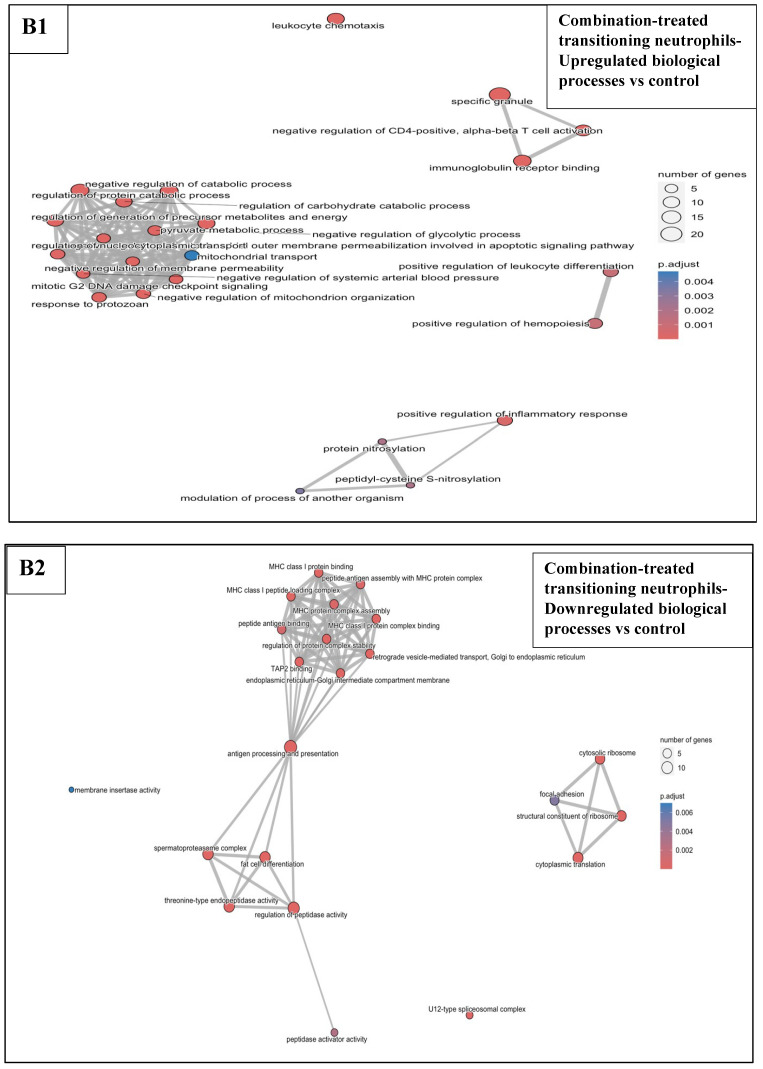

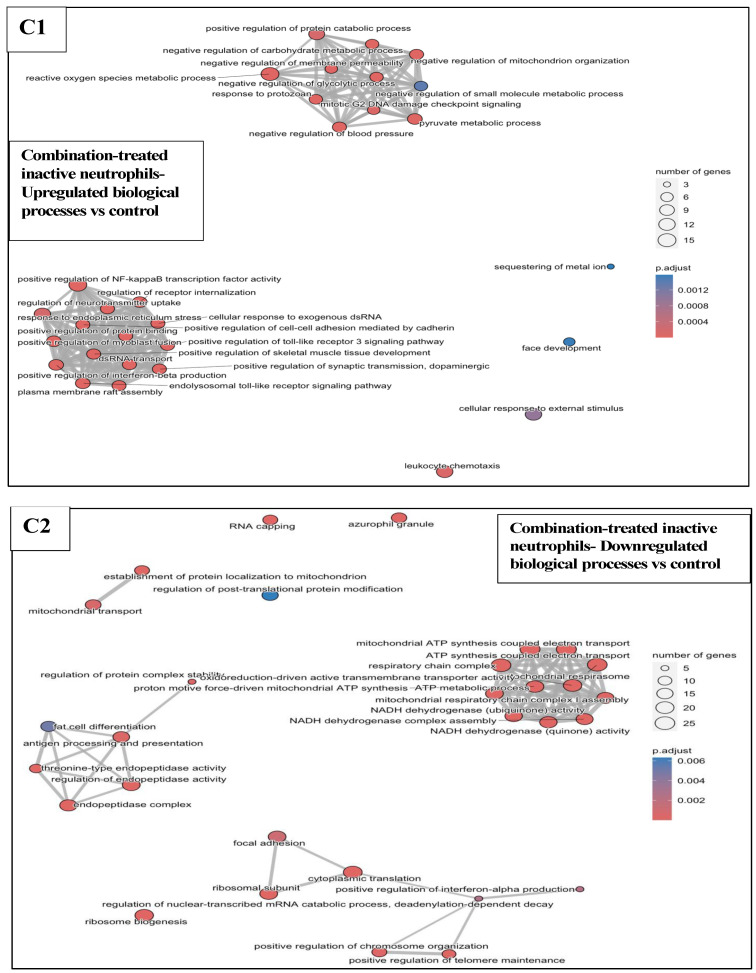
Functional enrichment analysis of DEG in the neutrophils of KPC tumours 24 h after treatment with the combined BH + reovirus compared to controls. KPC tumours were sham-exposed (*n* = 6) or treated with BH + reovirus (*n* = 4) and excised 24 h later. DEG results from the control vs. combined tumours were functionally analysed to illustrate the biological processes associated with DEG. These are shown in (**A1**,**A2**) for intermediate neutrophils, (**B1**,**B2**) for transitioning neutrophils, and (**C1**,**C2**) for inactive neutrophils. Results are shown for DEG with *p*-adjusted values < 0.05 between the combined and sham-exposed tumours and are presented as functional enrichment plots of these DEG. The key legends represent the number of Ensembl gene IDs by the size of the open circle, and the *p*-adjusted values show a significance range.

Twenty-four (24) hours after treatment of the KPC tumours, in active neutrophils, the combined treatments increased the transcription of genes (Figure A6E) associated with the NF-kβ transcription factor activity, TLR3 signalling, IFN-β production, myeloid leukocytes activation, and chemotaxis compared to the sham-exposed controls (Figure 8A1), whereas downregulated active neutrophil genes (Figure A6E) were associated with the TAP1 complex binding, antigen processing and presentation, and the regulation of T cell-mediated immunity (Figure 8A2). Some of these pathways were also modulated in the other major neutrophil clusters including the intermediate, transitioning, and inactive neutrophils (Figure A8). In macrophages, the overexpressed genes (Figure A6F) were associated with various innate immunity-related processes including type I IFN production, the MDA-5 signalling, the classical pathways of the complement activation, the clearance of apoptotic cells, TLR binding, and the NF-kB transcription factor activity (Figure 8B). In Th cells, the combination treatments led to increases in genes associated with type I IFN production and signalling and MDA-5 signalling in active Th (Figure 8C) and proliferating active Th (Figure 8D), and with the IFN-β production in the proliferating active Th (Figure 8D). In exhausted proliferating Tcyt upregulation of genes associated with the type I interferon (IFN) production, the MDA-5 signalling pathway, the negative regulation of virus replication, the regulation of the macrophage apoptotic process, the regulation of the adaptive immune response and the somatic recombination of immune receptors built from IgG superfamily domains were seen (Figure 8E). Finally, in B cells, positive regulation of type I IFN signalling pathways as well as genes associated with the IgG-mediated immune response and defence responses to virus infections were seen in the combination vs. sham-exposed controls (Figure 8F).

#### 3.6.4. Combination Treatments vs. Reovirus-Alone and BH-Alone Groups

**Figure A9:**
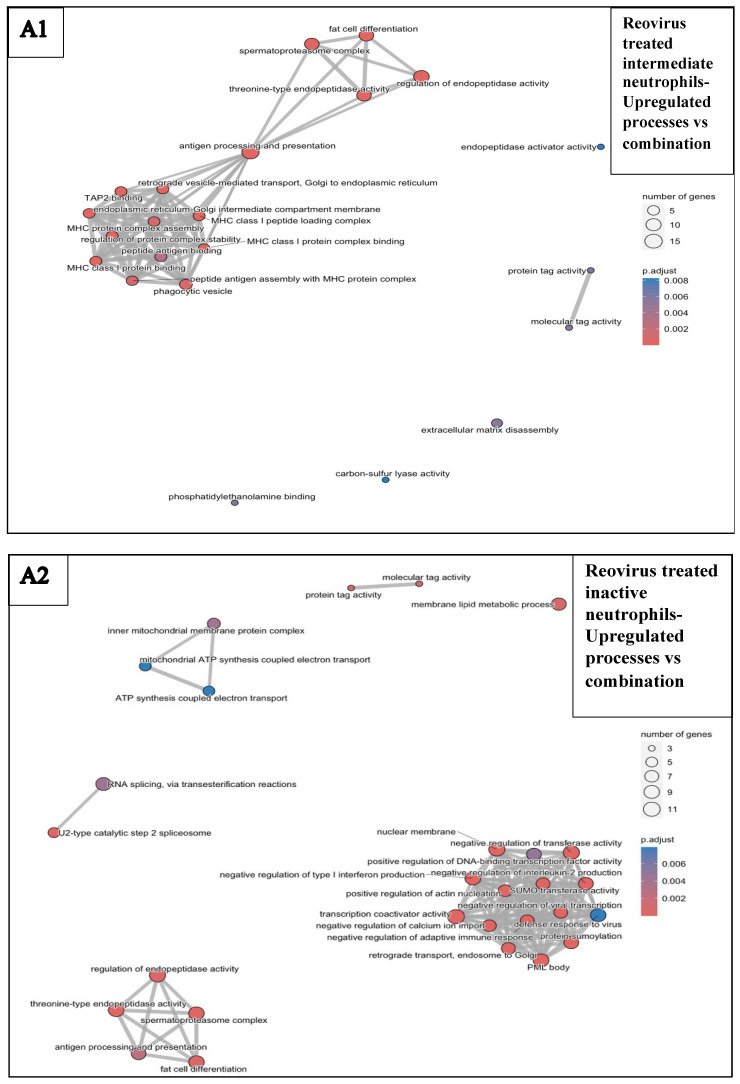

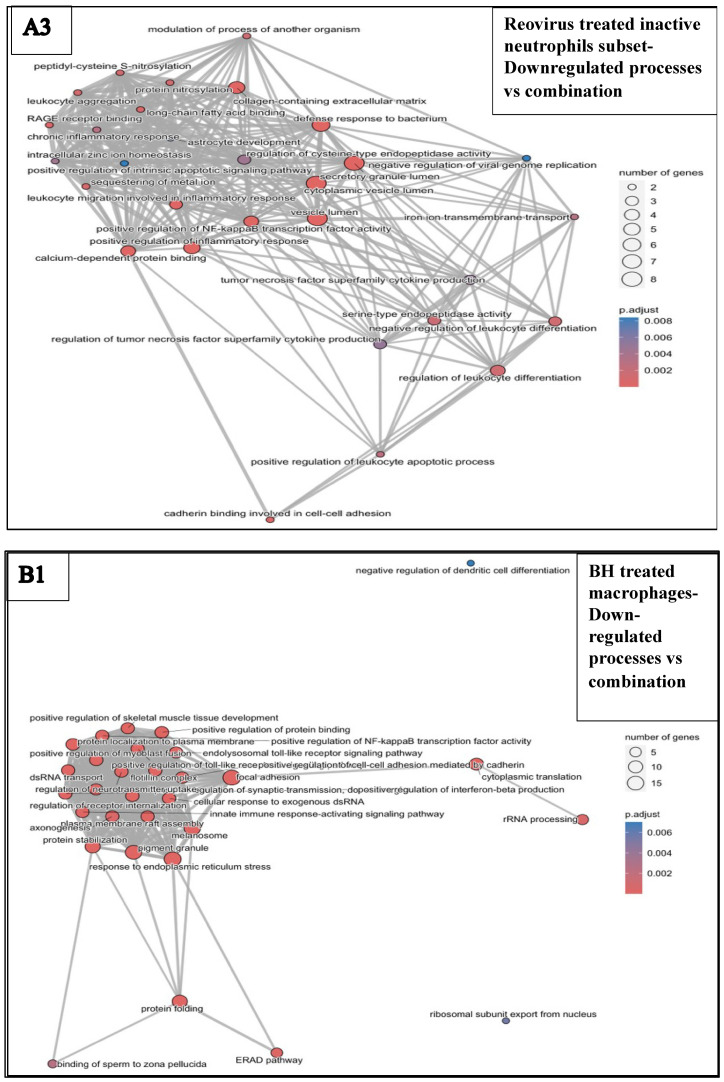

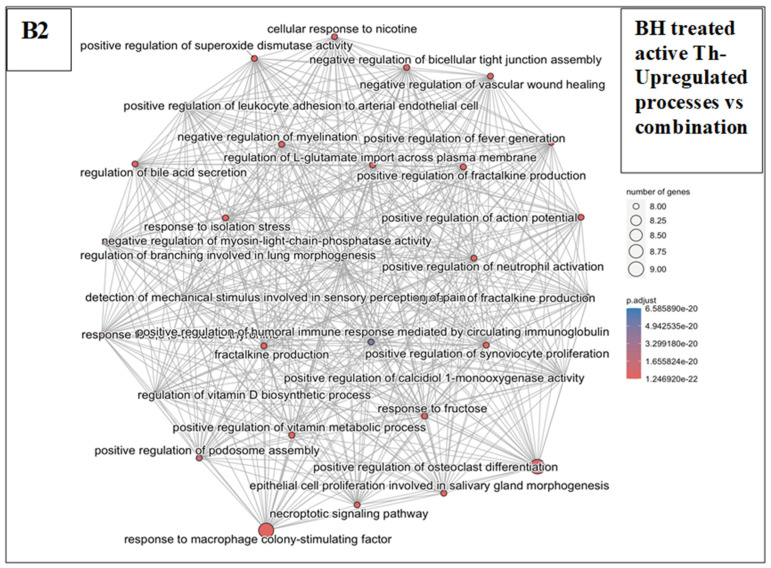
Functional enrichment analysis of DEG (*p*-adjusted values < 0.05) in KPC tumours 24 h after their exposure to reovirus alone (*n* = 6), BH alone (*n* = 5), or combined BH + reovirus (*n* = 4) treatments. Results are shown for the reovirus vs. combined treatments in (**A1**) for intermediate neutrophils, in (**A2**) for inactive neutrophils, in (**A3**) for the inactive neutrophils subset, and for the BH vs. combined treatments in (**B1**) for macrophages and in (**B2**) for active Th. Results are presented as functional enrichment plots. The key legends represent the number of Ensembl gene IDs by the size of the open circle, and the *p*-adjusted values show a significance range.

When combination treatments were compared to the reovirus-only treatments, the reovirus-treated intermediate neutrophils had higher levels of genes associated with the TAP2-dependent class I MHC complex (Figure A9A1). In inactive neutrophils, the reovirus treatments increased the expression of genes associated with the negative regulation of type I IFN production, the negative regulation of the adaptive immune responses and IL-2 production (Figure A9A2). In the inactive neutrophil subset, reovirus treatments decreased the expression of genes associated with the positive regulation of the NF-kβ activity and TNF superfamily cytokine production compared to the combination treatments (Figure A9A3). When the combination treatments were compared to the BH-only treatments, differences were seen in macrophages and active Th. In BH-exposed macrophages, downregulated genes were associated with the TLR and NF-kβ signalling, and responses to exogenous dsRNA (Figure A9B1), whereas in active Th, BH treatments upregulated genes that were associated with responses to the macrophage colony-stimulating factor (Figure A9B2). No significant differences were seen in the remaining cell types when the combination treatments were compared to the reovirus-only or the BH-only treatments.

## 4. Discussion

In this study, murine orthotopic KPC tumours were exposed to either reovirus alone, cavitation-inducing BH alone, or both (combination treatment). The histological appearance of the tumours was studied 6 and 72 h after the end of the BH or sham-BH treatments, and showed the destruction of parts of the tumour after ultrasound treatments compared to the sham-exposed and reovirus-treated tumours (Figure 2). The immune response of the subjects was investigated 24 h after treatment. All KPC tumours including those from control subjects contained immune cells that could create an anti-immune pro-tumour TME, including neutrophils overexpressing genes associated with an N2-like phenotype, CD8^+^ lymphocytes overexpressing immune checkpoint genes, and SIRPα^+^ macrophages (Figure 3 and Table 2 and Table A3). The modulation of the expression of thousands of immune cell genes in treated subjects compared to controls was demonstrated with the combination treatments, more than doubling the number of modulated genes compared to either the reovirus or BH treatments alone or when added together (Table 5). All treatments (reovirus alone, BH alone, and combination) increased the transcription of genes associated with the innate immune response, including type I IFN production and signalling (Figure 7, Figure 8 and Figure A7). In addition, reovirus increased gene expression associated with the viral defence responses (Figure A7), BH upregulated genes associated with TLR3 and NF-kβ signalling, but downregulated genes associated with the TAP complex (Figure 7). The latter was also seen after the combination treatments (Figure 8). The combined treatments increased the abundance of the active and total neutrophils (Figure 4A) and the NLR (Figure A3) in the tumours, which was not seen with the reovirus or BH-only treatments. Finally, ultrasound-driven decreases in the abundance of Treg led to a change in the immune architecture of the pancreatic TME, as demonstrated by the significant increases that were seen in the ratios of multiple cell subsets to Treg (Figure 5).

Extension to current knowledge: The immune effects of “thermal” and “mechanical” focused ultrasound in cancer have been investigated in the past by a number of research groups. Assessments have commonly used antibody-based histological analysis and flow cytometry, with few studies performing holistic transcriptional or proteomics studies. Consequently, little consensus exists about if and how an anti-cancer-specific immune response is activated after ultrasound treatments. Furthermore, some of the effects seen in pre-clinical studies in vivo, such as, for example, the abscopal effect, have not been convincingly replicated in the clinic. This necessitates a more detailed understanding of the immune mechanisms associated with focused ultrasound and immunotherapy treatments. Our study extends the depth of analysis shown in published studies [27] including our own [24]. Previously, the activation of components of the immune system was demonstrated in the Pan02 murine subcutaneous pancreatic cancer model (which carries no KRAS or P53 mutations) using flow cytometry and bulk RNA sequencing (Qiagen “Cancer Inflammation and Immunity Crosstalk” SuperArray platform). Hendricks-Wenger and colleagues reported the upregulation of 8/100 immune genes 24 h after cavitation cloud histotripsy exposures, using a 1 MHz, eight-element histotripsy transducer generating short pulses of <2 cycles, at a prf of 250 Hz and an exposure time of 1 sec, hence sending 250 shots at each treatment point. Upregulated genes included the pro-inflammatory CCR7, CXCL9, CXCR4, GZMB, IL13, IL22, IL5, and HSP90AB1 genes. These initial increases were followed by upregulation of anti-inflammatory genes being seen at the survival endpoints, days later. Of the eight immune cell types investigated using flow cytometry at three different time points (1, 7, and 14 days after treatment), including granulocytes (CD45^+^CD11C^−^Ly6C^−^Ly6G^+^ cells), statistically significant modulation of macrophages, DC, and Treg was seen 14 days after treatment, and of Th 7 days after exposure [27]. In our study, a reduction in the transcription of GZMB in the macrophages and DC in the tumours of BH-treated subjects compared to the sham-exposed controls was seen. The two forms of histotripsy (boiling and cavitation cloud) use the same fundamental mechanism of tissue damage (collapsing bubbles), so these results demonstrate the need for detailed studies to investigate thoroughly their immune mechanisms. Also, we used an orthotopic tumour model based on KPC cells (with G12D KRAS and TP53 mutations) injected into the pancreas of subjects to replicate the native environment in which pancreatic tumours grow. Although the anatomical features differ between humans and mice (e.g., human PDACs typically extend beyond the organ and intertwine with major veins and arteries), the KPC mutations are among the most frequently seen in human PDACs, with KRAS present in >90% and p53 mutations present in >50% of human PDACs. The murine KPC tumours also have a dense collagen stroma [28] and compete with the neighbouring normal pancreas and other organs for nutrients and resources.

Franks and colleagues have published a series of studies on the effects of mechanical therapy ultrasound using a relatively low P- of 6 MPa (below the range accepted as inducing BH, 10–20 MPa [23]) to treat murine breast cancer and melanoma. Bulk proteomic and transcriptomic approaches were used that showed transcriptome and proteome modulation in the ultrasound-treated tumours which depended on the tumour type [29,30,31]. Abe et al. used focused ultrasound at 1.5 MHz, 2% dc, 200 W, and a prf of 5 Hz, for 20 s treatments in a HER2-transduced preclinical murine breast cancer model to induce mechanical damage with or without the addition of anti-PDL1 (given intraperitoneally 3 and 6 days after the ultrasound treatment), and investigated the transcription of genes 8 days after exposures using single-cell transcriptomics [32]. In CD8^+^ T cells, all treatments (ultrasound only, PDL-1 only, and combination) increased the expression of genes associated with type I IFN-mediated signalling, activated T-cell proliferation, and promoted chemokine and cytokine secretion compared to untreated controls. Genes related to CD8^+^ T cell activation including CD33, CX3CR1, CXCL3, CXCL11, and CXCL16 were overexpressed >2-fold in the combination treatment group (ultrasound and anti-PDL1) compared to the group using mechanical ultrasound therapy alone, providing a possible explanation for the enhanced anti-tumour effects that were observed for the combination vs. ultrasound treatment group in the same study. The use of non-transduced “immune cold” pre-clinical cancer models should provide further data about the effects and mechanisms associated with the ultrasound and anti-PDL1 treatments.

BH-induced acoustic cavitation and histological assessments: Acoustic cavitation detection of broadband emissions is generally accepted to indicate the violent collapse of clouds of oscillating bubbles (inertial cavitation), and the release of energy that could lead to tumour disruption. This is a stochastic process, and as such, bubble clouds with unpredictable numbers of bubbles can be created upon ultrasound exposure, and the energy released from each bubble can vary significantly. Tissue architecture in and around the target volume will also affect this process, resulting in some tumour exposure positions having a higher propensity for acoustic cavitation than others. As a result, identical ultrasound exposure pulses are unlikely to produce identical acoustic cavitation outcomes when delivered repeatedly to a single location or different positions within a target tumour. Instead, they can create a range of inertial cavitation activity from undetectable levels, up to levels high enough to saturate detection equipment.

In this study, the number of broadband-positive pulses and the appearance of hyperechoic regions after treatment with BH were used as indicators of the presence of acoustic cavitation activity (both shown in Table 1). Ultrasound treatment of the KPC tumours resulted in detectable broadband signals during the treatment of every tumour. Hyperechoic region formation was also seen on real-time ultrasound images in all tumours except one which had an unusually bright background echogenicity within the tumour before treatment. Histological evidence indicated the destruction of ultrasound-treated tumours by ultrasound-induced acoustic cavitation. A tumour architecture composed of intact, densely packed nuclei was seen within the cells of tumours in the sham and reovirus-treated groups (Figure 2A–C), whereas enlarged intercellular spaces and oddly shaped nuclei were seen in KPC tumours at our first assessment time point 6 h after ultrasound treatments (Figure 2D1,E2). By 72 h, the disrupted zones of the ultrasound-treated tumours had developed into necrotic-like regions with few (or no) intact KPC cells (Figure 2F2,G2), suggesting that extensive BH-induced cell death had taken place in the core of the tumours.

Cluster annotation: PDAC tumours have been considered to be immune “cold” tumours with few immune cells compared to, for example, melanomas. Here we have demonstrated that the murine KPC TME has a diverse array of immune cell infiltrates associated with it (Figure 3 and Figure A2). To understand the role of these infiltrates, they first had to be identified as precisely as possible, and for this reason the UMAP clusters were annotated manually using conserved biomarkers for each immune cell type. Annotation decisions were based on the following hallmarks:

Neutrophils: Seven (7) **neutrophil subsets** were distinguished (Table 2A,B). They all expressed various protein levels of Ly6G (Figure A2D), overexpressed the genes ITGAM [33], FCGR3 [34], SELL, S100A8, and IFITM1 and were negative for CCL2 differentiating them from macrophages. Also, they expressed the CD14 gene which is expressed by neutrophils under inflammatory conditions [35,36]. All neutrophil subsets showed an N2-like (pro-tumour growth, immune suppressive) gene expression pattern by overexpressing ENTPD1 [37], CXCR2 [33], S100A9 [38], IL10R [39,40], CD177 [41,42], OSM [43], TGFβR [44] (all but the active neutrophils subset cluster), PDL1, and MMP9 [45], and by downregulating pro-inflammatory genes like NOX2 [46], IFN-γ, and IFN-γR that are commonly associated with an N1-like phenotype. All neutrophil clusters overexpressed the activation marker gene CD63 [47], TLRs, and TNF (Table 2A). These seven clusters were further separated into four large and three small clusters. The first of the large clusters was the **activated-neutrophil** cluster because these cells increased the transcription of genes associated with neutrophil activation including MMP8, a neutrophil degranulation marker [48], several INF-γ-inducible genes including CXCLL10, IFITM6, IFIT2, and TNFAIP6 which have been associated with the activation of innate immune response and M2 macrophage polarisation [49], and MRGPRA2B, the protein product of which acts as a detector of foreign elements on the surface of neutrophils (Table 2B). Another large neutrophil cluster was the **intermediate-neutrophil** cluster because, here, the transcription of activation genes was lower than that seen in the activated neutrophils cluster (e.g., MMP8, IFN-γ-responsive genes) or not expressed at all (TNFAIP6, MRGPRA2B, CXCL10 [33]) (Table 2B). The third large neutrophil cluster was annotated as the **inactive-neutrophils** because it showed no overexpression of all the above-mentioned biomarkers (Table 2B). In addition, three smaller clusters were distinguished. The cells in the **active-neutrophils subtype** cluster expressed similar biomarkers to the active neutrophils including several IFN-stimulated genes (e.g., IFITM6, IFIT2, IFIT3, IFIT1) and MMP8, but did not overexpress CXCL10 and Dach1 (Table 2B). The **intermediate-neutrophil subtype cluster** had a similar expression pattern to the intermediate neutrophils, and in addition, expressed high levels of the SIGLECF gene (Table 2B). Neutrophils that overexpress SIGLECF have been shown to promote tumour growth and the suppression of the immune system [50]. The **inactive-neutrophil subtype** had a similar expression pattern to the inactive neutrophils lacking the overexpression of the IFN-stimulated genes, CXCL10, TNFAIP6, and MMP8. Again, this cluster showed high overexpression of SIGLECF and IL23A; the latter was not seen in the inactive-neutrophil cluster (Table 2B). Finally, the **transitioning-neutrophil** cluster was identified as such due to its location in the UMAP and the overexpression of genes associated with both the active and intermediate neutrophils. Annotation decisions for all remaining clusters are discussed in the manual annotation discussion section of Appendix A.

Collectively, these cluster annotation results demonstrate the existence of a KPC TME that is hostile to the host by favouring tumour growth and the regulation of the anti-cancer immune response in some ways: Firstly, neutrophils, key cells involved in the innate immune response, exhibited N2-like gene expression patterns which are generally associated with pro-tumourigenic processes, whereas N1-like neutrophils would favour a pro-inflammatory phenotype. Secondly, the majority of the Tcyt overexpress exhaustion marker genes such as the immune checkpoints CTLA4, PDCD1, LAG3, TIM, and TIGIT suggestive of the regulation of their cytolytic potential. Thirdly, the presence of a distinct cluster of SIRPα^+^ macrophages suggests that at least some phagocytic cells would be prone to stimulation by tumour-expressed CD47, thereby making them incapable of engulfing abnormal cellular bodies and limiting their anti-tumour role [51,52].

Changes in immune cell abundance: Compared to the sham-exposed group, reovirus treatments decreased the abundance of macrophages (Figure 4B), whereas BH increased transition neutrophils (Figure 4A) and killed Treg (Figure 4C). Combination treatments decreased both the macrophages (Figure 4B) and Treg (Figure 4C) similarly to the reovirus-only and BH-only treatments, respectively. In addition it increased the abundance of active and total neutrophils **(**Figure 4A) and the NLR which has been postulated as a blood marker of poor prognosis [53] (Figure A3). These results suggest that the combined treatments activate the innate immune response, more than the reovirus and BH treatments when used alone, by increasing the infiltration of the tumours by neutrophils. But, ultimately, this may prove to be a “double-edged sword”. The overexpression of several immune-regulatory genes by these neutrophils suggests that their recruitment to the pancreatic TME may regulate the further activation of the immune response. Experiments designed to block these neutrophils would shed more light on their role in the response of the tumours to the BH and reovirus treatments.

Activated Tcyt and NK cells attack and kill cells that are recognised as foreign, including reovirus-infected and tumour cells. In this study, exposure of the KPC tumours to the combined treatments increased the ratio of the NK cells (Figure 5B), exhausted Tcyt and proliferating Tcyt (Figure 5D) to Treg compared to the sham-exposed controls. It is thus reasonable to suggest that these cells have the potential to induce cell death 24 h after treatment independently of the regulatory functions of Treg. Also, the increases seen in the ratio of other pro-immune cells to Treg such as the DC (Figure 5B), inflammatory monocytes (Figure 5B), and active and proliferating active Th (Figure 5C) are likely to create a more favourable immune TME in the combination-treated tumours compared to the sham-exposed tumours.

Functional enrichment analysis—All treatments: Treatment of the KPC tumours with reovirus or BH resulted in the overexpression of genes associated with the activation of the innate immune response.

Reovirus treatments modulated the transcription of genes in cells of monocytic lineage (macrophages and inflammatory monocytes) whereas only modest DEG was seen in the remaining clusters (Table 5). In macrophages, upregulated genes including SIGLEC1, DHX58, XAF1, IRF7, OAS3, IFIH1, OAS2, TLR3, MORC3, OTUD5, PARP9, STAT2, SP100, USP18, and ADAR (Figure A6A) have been associated with type I IFN signalling and production (Figure A7). Type 1 IFNs include the IFN-α and IFN-β cytokines which play key roles as communication signals for the activation of the innate immune responses against the viral infections. Genes associated with viral defence responses and/or the classical complement activation pathway (Figure A7A1), for example C4B, CLU, RTP4, ZNFX1, DDX60, PML, IFIT3, DTX3L, MX1, EIF2AK2, IRF9, IL15, BST2, MLKL, and IFIT1 were also observed to be overexpressed (Figure A6A), suggesting the central role of these cells in the clearance of the virally infected cells.

BH treatments significantly modulated gene expression in active and intermediate neutrophils, whereas the downregulation of genes was seen in macrophages and DC (Table 5). In active neutrophils, overexpressed genes including IL1RAP, ADAM8, TLR4, CX3CR1, FLOT1, and IL18 (Figure A6B) have been associated with NF-kβ and TLR3 signalling (Figure 7A1,B). NF-kβ is a multifaceted transcription factor that coordinates the inflammatory response and the response of cells to cellular stress. TLR3 signalling is associated with the response of cells to double-strand (ds)RNA. It is reasonable to assume that the BH-destroyed KPC and other stroma cells would be the source of this dsRNA. On the other hand, in macrophages, BH treatments downregulated genes including IL27RA, CD3E, CD96, CD226, CD2, and PGLYRP1, which have been associated with the regulation of type II IFN production, and GZMB, SH2D1A, NKG7, LAG3, CTLA4, TNFSF11, CAMK2N1, ETS1, LILRA5, and IFNG (Figure A6C) that have been associated with the regulation of the lymphocyte and NK cell-mediated immunity (Figure 7C). These processes would be expected to play a role in a cancer-specific adaptive immune response. In DC, downregulated genes including CD160, NKG7, CD8A, ITK, TRBC1, TRBC2, LCK, CD3D, CD3G, CD3E, and CD28 (Figure A6D) have been associated with the T cell and NK cell immune response and the class I MHC (Figure 7D), wheras in active neutrophils BH treatments downregulated genes such as TAP2, PSMB8, H2.Q7, and H2.K1 (Figure A6B), which have been associated with the class I MHC and TAP complexes (Figure 7A2). The class I MHC plays a key role in the presentation of intracellular antigens/peptides to cytotoxic T cells that would then be expected to elicit a sustained adaptive immune response against these antigens. This process is helped by the TAP complex which is responsible for transporting peptides from the cytosol to the cell surface so that they are then presented by the MHC. These results suggest that while BH activated at least some parts of the innate immune response, at the same time it downregulated components associated with the adaptive immune response in various cells. In this context, Price, Bullock and colleagues have recently shown, in a pre-clinical melanoma B16-F10 model using 3 ms BH exposures of P^−^ = 21 MPa, duration of 10 s, prf = 4 Hz, spacing every 1mm, treatment planes separated by 2mm, the release of free antigen into lymph vessels and its acquisition by antigen-presenting cells within 24 h of treatment [54], thus providing at least one way by which the effects of BH on some antigen presentation processes in the pancreatic TME, as seen in this study, could be overcome.

The combination treatments resulted in the modulation of a much higher number of genes compared to the reovirus-only and BH-only treatments in cells of granulocytic (active, intermediate, transitioning, and inactive neutrophils) and monocytic origin (macrophages, inflammatory monocytes, and MDSC-enriched cells) (Table 5). Overexpressed genes in active neutrophils (e.g., S100A9, S100A8, ADAM8, CFLAR, TLR4, CX3CR1, TRAF1, IL1RAP, and FLOT1) (Figure A6E) have been associated with the NF-kβ and TLR3 signalling (Figure 8A1) as was seen for the BH-alone treatments. Overexpressed genes in macrophages (e.g., C4B, SIGLEC1, IRF7, TLR3, DHX58, XAF1, OAS2, DDX60, TLR6, and ZNFX1) (Figure A6F) have been associated with the complement activation and type I IFN signalling and production (Figure 8B), as was seen for the reovirus-only treatments. The combined treatments also increased genes that have been associated with the MDA5 signalling pathway in various cell types (Figure 8) including DDX60, IRF7, DHX58, and OAS3 (Figure A6F). This pathway is an anti-viral immune response mechanism that leads to the production of type I IFNs which would then elicit downstream immune processes. IFN-responsive genes that have been shown to play a role in the host’s anti-viral defences and that were upregulated by the combined treatments in active, intermediate, transitioning and inactive neutrophils included IFITM6 [55], IFI27L2A [56], IL15 [57], and CD14 [58] (Figure 6B). Additionally upregulated genes included the neutrophil and lymphocyte chemoattractants CXCL3 [59], CXCL2 [60], and CCR1 [61], and PLAUR which protects cells against viruses [62] and facilitates cell migration [63] thus showing one mechanism by which neutrophils would have been attracted in the combination-treated KPC TME (Figure 4A). In response to the combination treatments neutrophils also upregulated genes which are associated with overcoming cell stress such as IER-3, an apoptosis inhibitor that protects cells from TNFα induced apoptosis [64] and HILPDA which enables cells to overcome hypoxia [65]. Neutrophil genes associated with a pro-tumour growth phenotype were also upregulated after the combination treatments compared to controls, including the CCL6 gene which promotes immunosuppressive networks, including anti-immune M2 macrophage polarisation [66], and tumour progression [67,68] and LILRB4 that has been associated with negative regulation of the immune response, antigen capture and presentation, and is a potential immunotherapy target [69,70]. These are not unexpected results considering that the neutrophils in the KPC TME express N2-like transcriptomic signatures, as shown earlier in this study (Table 2). The two commonly upregulated genes in the active neutrophils, macrophages, active proliferating Th, and exhausted proliferating Tcyt included the gene XAF1, a tumour suppressor that is upregulated in virus-infected cells and enhances IFN-induced apoptosis [71,72], and PYDC4, which is predicted to regulate the activation of the inflammasomes, which are intracellular bodies that coordinate the response of cells to viral infections [73] (Figure 6C). These results suggest that the combined treatments activate the innate immune response to resolve the reovirus infection and to limit the damage caused to the cellular structures by the BH treatments, but also attract to the tumour immune cells like neutrophils that, in this tumour model, show a pro-tumour N2-like phenotype.

Compared to the sham-exposed controls, the combined treatments, downregulated active neutrophil genes including TAP2, PSMB8, TAPBPL, ATP2A3, HSPD1, ZBTB1, ERAP1, PSME1, and CTSS (Figure A6E) that have been associated with antigen processing and presentation, and the regulation of T cell-mediated immunity (Figure 8A2). Some of these genes were downregulated across neutrophil subsets, for example, PSMB8 and TAPBP (Figure 6A). PSMB8 directs the creation of immunoproteasomes which distinguish and degrade foreign proteins eg, those originating from viruses [74]. TAPBP plays a role in the presentation of cancer cell antigens by antigen-presenting cells [75]. These results suggest that the downregulation of the TAP-dependent class I MHC, at this early time point, is likely an ultrasound-driven event as described above for the BH-only treatments (Figure 7A2). We hypothesise that this could benefit the host by reducing the number of intracellular self-antigens exposed on the class I MHCs of the ultrasound-treated tumours, thereby reducing the risk of an immune response being mounted against self-antigens.

Study limitations: This study has used only one time point for the single-cell immune analysis (24 h). The primary reason for this is that our previous study [24], which investigated the immune response in PDAC at longer time points after treatment (12-day assessment for CD8^+^ and CD4^+^ cells), showed limited beneficial effects on the ultrasound-treated PDAC tumour growth and immune response at that late stage. To understand those diminishing effects, the 24 h time point was used here as a reasonable indicator of the innate immune response activation phase. Another limitation of this study is that the KPC cells were not investigated. This is because there is a trade-off between the depth of analysis possible with current single-cell technologies, cost, and the number of samples assessed. To achieve unparalleled sequencing depth, we chose to concentrate our investigation on the repertoire of the tumour-associated immune cells and to investigate each tumour as a unique sample, thereby providing a better illustration of the variability seen between and within treatment groups. Finally, in future studies, transcriptomic results will need to be complemented by protein and enzyme activity data to fully validate the various cell subtypes and their regulation seen in this study.

## 5. Conclusions

In this study, the acute immune response of the orthotopic murine pancreatic KPC tumours 24 h after treatment with reovirus, cavitation-inducing BH (boiling histotripsy), and the combination of BH and reovirus was compared to both sham-exposed controls and each other. Our data shows that (a) the pancreatic TME consists of immune cell subsets programmed to create a hostile environment for the host TME by overexpressing immune signatures that are associated with an N2-like phenotype in neutrophils, an exhaustion phenotype in cytotoxic lymphocytes, and an inactivation phenotype in a macrophage subset; (b) all treatments overexpressed genes associated with the activation of the innate immune response compared to the sham-exposed controls, with the effects being more marked after the combined treatments which showed higher levels of tumour-infiltrating neutrophils and higher modulation of gene expression in the immune cells than the reovirus and BH treatments used alone; (c) the ultrasound-based treatments (BH and combination) downregulated genes associated with antigen presentation, possibly as a mechanism to limit activation of the immune system against self-antigens; and (d) BH and combination treatments changed the balance of pro-immune cells to Treg in the pancreatic TME, thereby creating a more favourable pro-immune tumour micromilieu. These results demonstrate that the combined BH and reovirus treatment enhances the immune response compared to the treatments used alone. They also show some of the potential mechanisms by which the immune TME can facilitate the immune evasion capabilities of pancreatic tumours. In future studies, the BH and reovirus treatments will be combined with immunotherapies (e.g., neutrophil depletion) to investigate the effects of these cells on pancreatic tumour growth.

## Figures and Tables

**Table 1:** Acoustic cavitation detection data during and after BH and combination (BH + reovirus) treatments of KPC tumours in every subject. Colour coding has been used to facilitate visual assessment of the numerical data. Each column is colour-coded from lowest value (white) to midpoint (orange) and maximum value (red) automatically in Excel (except for the percentage of HH positive pulses which are all high and therefore coded red).

Subject	Number of Exposure Positions per Tumour	Number of Exposure Pulses per Tumour	Percentage of HH-Positive Exposure Pulses (%)	Number of HH Positive Exposure Pulses per Tumour	Percentage of Broadband Signal Exposure Positive Pulses (%)	Number of Broadband-Positive Exposure Pulses per Tumour	Hyper-Echoic Signals
BH- Subject 1	28	660	98	650	14	95	Yes
BH 2- Subject 2	17	350	100	350	94	330	Yes
BH 3- Subject 3	30	720	100	720	20	150	In-conclusive
BH 4- Subject 4	19	450	100	450	77	345	Yes
BH 5- Subject 5	25	535	100	535	67	360	Yes
Comb 1- Subject 1	13	310	97	300	100	310	Yes
Comb 2- Subject 2	10	230	91	210	95	220	Yes
Comb 3- Subject 3	22	530	100	530	4	20	Yes
Comb 4- Subject 4	17	350	100	350	2	6	Yes

**Table 2:** Manual annotation of immune cell clusters observed in KPC tumours. Thirty (30) cell clusters were identified in the UMAP of the KPC tumours used in this study. These clusters were annotated after assessing conserved gene expression within each cluster. These conserved genes are listed for neutrophils (Table 2A,B), CD4^+^ lymphocytes (Table 2C), and CD8^+^ lymphocytes (Table 2D). Gene overexpression in a cluster was considered “high” if their AvgLog_2_Fc > 1 (dark green), “moderate” if 0.5 < AvgLog_2_Fc < 1 (light green), “low” if 0.25 < AvgLog_2_FC < 0.5 (amber), and “negative” (neg) if AvgLog_2_FC < 0.25 (red).

**A**—**Neutrophils**																																																		
**Clusters**	Relative expression of annotation genes—**AvgLog_2_Fc > 1** (dark green), **0.5 < AvgLog_2_Fc < 1** (light green), **0.25 < AvgLog_2_FC < 0.5** (amber), **AvgLog_2_FC < 0.25** (red).																																																	
Transitioning	CD63	ITGAM		FCGR3	SELL	ENTPD1			PDL1		CYBB		CD177			CXCR2			MMP9		IL10R			OSM	TGF-βR			IFNγ^/^IFNγR			TLR4/CD14				TLR2			CCL2		S100A8 S100A9				TNF				CD24A		
Active	CD63	ITGAM		FCGR3	SELL	ENTPD1			PDL1		CYBB		CD177			CXCR2			MMP9		IL10R			OSM	TGF-βR			IFNγ^/^IFNγR			TLR4/CD14				TLR2			CCL2		S100A8 S100A9				TNF				CD24A		
Active subset	CD63	ITGAM		FCGR3	SELL	ENTPD1			PDL1		CYBB		CD177			CXCR2			MMP9		IL10R			OSM	TGF-βR			IFNγ^/^IFNγR			TLR4/CD14				TLR2			CCL2		S100A8 S100A9				TNF				CD24A		
Intermediate	CD63	ITGAM		FCGR3	SELL	ENTPD1			PDL1		CYBB		CD177			CXCR2			MMP9		IL10R			OSM	TGF-βR			IFNγ^/^IFNγR			TLR4/CD14				TLR2			CCL2		S100A8 S100A9				TNF				CD24A		
Intermediate subset	CD63	ITGAM		FCGR3	SELL	ENTPD1			PDL1		CYBB		CD177			CXCR2			MMP9		IL10R			OSM	TGF-βR			IFNγ^/^IFNγR			TLR4/CD14				TLR2			CCL2		S100A8 S100A9				TNF				CD24A		
Inactive	CD63	ITGAM		FCGR3	SELL	ENTPD1			PDL1		CYBB		CD177			CXCR2			MMP9		IL10R			OSM	TGFbR			IFNγ^/^IFNγR			TLR4/CD14				TLR2			CCL2		S100A8 S100A9				TNF				CD24A		
Inactive subset	CD63	ITGAM		FCGR3	SELL	ENTPD1			PDL1		CYBB		CD177			CXCR2			MMP9		IL10R			OSM	TGF-βR			IFNγ^/^IFNγR			TLR4/CD4				TLR2			CCL2		S100A8 S100A9				TNF				CD24A		
**B**—**Neutrophils**																																																		
**Clusters**	Additional annotation genes. Relative expression—**AvgLog_2_Fc > 1** (dark green), **0.5 < AvgLog_2_Fc < 1** (light green), **0.25 < AvgLog_2_FC < 0.5** (amber), **AvgLog_2_FC < 0.25** (red).																																																	
Active	MMP8	IFIT2	IFITM6	CXCL10	CXCL3	CCL4				CCL6		IFIT3		IFIT3B			IFITM3			IFIT1		ISG20		IFIT1BL2		TNFAIP6			DACH1			MRGPRA2B				GSTM1			IL23A		SIGLECF				LTF		ADAM8		ADAMDEC1	
Active subset	MMP8	IFIT2	IFITM6	CXCL10	CXCL3	CCL4				CCL6		IFIT3		IFIT3B			IFITM3			IFIT1		ISG20		IFIT1BL2		TNFAIP6			DACH1			MRGPRA2B				GSTM1			IL23A		SIGLECF				LTF		ADAM8		ADAMDEC1	
Intermediate	MMP8	IFIT2	IFITM6	CXCL10	CXCL3	CCL4				CCL6		IFIT3		IFIT3B			IFITM3			IFIT1		ISG20		IFIT1BL2		TNFAIP6			DACH1			MRGPRA2B				GSTM1			IL23A		SIGLECF				LTF		ADAM8		ADAMDEC1	
Intermediate subset	MMP8	IFIT2	IFITM6	CXCL10	CXCL3	CCL4				CCL6		IFIT3		IFIT3B			IFITM3			IFIT1		ISG20		IFIT1BL2		TNFAIP6			DACH1			MRGPRA2B				GSTM1			IL23A		SIGLECF				LTF		ADAM8		ADAMDEC1	
Transitioning	MMP8	IFIT2	IFITM6	CXCL10	CXCL3	CCL4				CCL6		IFIT3		IFIT3B			IFITM3			IFIT1		ISG20		IFIT1BL2		TNFAIP6			DACH1			MRGPRA2B				GSTM1			IL23A		SIGLECF				LTF		ADAM8		ADAMDEC1	
Inactive	MMP8	IFIT2	IFITM6	CXCL10	CXCL3	CCL4				CCL6		IFIT3		IFIT3B			IFITM3			IFIT1		ISG20		IFIT1BL2		TNFAIP6			DACH1			MRGPRA2B				GSTM1			IL23A		SIGLECF				LTF		ADAM8		ADAMDEC1	
Inactive subset	MMP8	IFIT2	IFITM6	CXCL10	CXCL3	CCL4				CCL6		IFIT3		IFIT3B			IFITM3			IFIT1		ISG20		IFIT1BL2		TNFAIP6			DACH1			MRGPRA2B				GSTM1			IL23A		SIGLECF				LTF		ADAM8		ADAMDEC1	
**C**—**CD4^+^ lymphocytes**—Relative expression of annotation genes—**AvgLog_2_Fc > 1** (dark green), **0.5 < AvgLog_2_Fc < 1** (light green), **0.25 < AvgLog_2_FC < 0.5** (amber), **AvgLog_2_FC < 0.25** (red).																																																		
**Clusters**	Marker genes				Activation genes															Immune checkpoint genes													Proliferation genes								Various genes									
Naïve/ inactive Th	FOXP3	CD3e	CD4	FR4	CD25	CD69		TNFRSF9		TNFRSF4					CCR5			IFNγ		CTLA4			PDCD1	LAG3	TIM3		TIGIT			ICOS		MKI67		TOP2A			PCNA		CD40LG			TBET							IL7R	
Active Th	FOXP3	CD3e	CD4	FR4	CD25	CD69		TNFRSF9		TNFRSF4					CCR5			IFNγ		CTLA4			PDCD1	LAG3	TIM3		TIGIT			ICOS		MKI67		TOP2A			PCNA		CD40LG			TBET							IL7R	
Proliferating active Th	FOXP3	CD3e	CD4	FR4	CD25	CD69		TNFRSF9		TNFRSF4					CCR5			IFNγ		CTLA4			PDCD1	LAG3	TIM3		TIGIT			ICOS		MKI67		TOP2A			PCNA		CD40LG			TBET							IL7R	
Treg	FOXP3	CD3e	CD4	FR4	CD25	CD69		TNFRSF9		TNFRSF4					CCR5			IL10		CTLA4			PDCD1	LAG3	TIM3		TIGIT			ICOS		MKI67		TOP2A			PCNA		CD40LG			KLRG1/CCR2					ITGAE		IL7R	
Tfh	FOXP3	CD3g	CD4	FR4	CD25	CD69		TNFRSF9		TNFRSF4					CCR5			IL10		CTLA4			PDCD1	LAG3	TIM3		TIGIT			ICOS		MKI67		TOP2A			CCNA2		CD40LG			IL10/FCGR3					TNF		IL7R	
**D**—**CD8^+^ lymphocytes**—Relative expression of annotation genes—**AvgLog_2_Fc > 1** (dark green), **0.5 < AvgLog_2_Fc < 1** (light green), **0.25 < AvgLog_2_FC < 0.5** (amber), **AvgLog_2_FC < 0.25** (red).																																																		
**Clusters**	Lineage genes				Activation genes									Immune checkpoint genes											Proliferation/cell cycle genes										Transcription factor genes								Various genes							
Senescent Tcyt	KLRG1	CD3e	CD8α	GZMB	FASL		CD69		TNFRSF9			PFN		LAG3			PDCD1			CTLA4			TIGIT	TIM3	MKI67			TOP2A			PCNA		CCNA2		TBET			TOX		EOMES			IFNγ			SELL			IL7R	CD44
Exhausted proliferating Tcyt	KLRG	CD3e	CD8α	GZMB	FASL		CD69		TNFRSF9			PFN		LAG3			PDCD1			CTLA4			TIGIT	TIM3	MKI67			TOP2A			PCNA		CCNA2		TBET			TOX		EOMES			IFN-γ			SELL			IL7R	CD44
Terminally Exhausted Tcyt	KLRG	CD3e	CD8α	GZMB	FASL		CD69		TNFRSF9			PFN		LAG3			PDCD1			CTLA4			TIGIT	TIM3	MKI67			TOP2A			PCNA		CCNA2		TBET			TOX		EOMES			IFNγ			SELL			IL7R	CD44
Exhausted Tcyt	KLRG	CD3e	CD8α	GZMB	FASL		CD69		TNFRSF9			PFN		LAG3			PDCD1			CTLA4			TIGIT	TIM3	MKI67			TOP2A			PCNA		CCNA2		TBET			TOX		EOMES			IFNγ			SELL			IL7R	CD44
Tcyt subset 1	KLRG	CD3e	CD8α	GZMB	FASL		CD69		TNFTSF9			PFN		LAG3			PDCD1			CTLA4			TIGIT	TIM3	MKI67			TOP2A			PCNA		CCNA2		TBET			TOX		EOMES			IFNγ			SELL			IL7R	CD44
Tcyt subset 2	KLRG	CD3e	CD8α	GZMB	FASL		CD69		TNFTSF9			PFN		LAG3			PDCD1			CTLA4			TIGIT	TIM3	MKI67			TOP2A			PCNA		CCNA2		TBET			TOX		EOMES			IFNγ			SELL			IL7R	CD44
Proliferating Tcyt	KLRG	CD3e	CD8α	GZMB	FASL		CD69		TNFTSF9			PFN		LAG3			PDCD1			CTLA4			TIGIT	TIM3	MKI67			TOP2A			PCNA		CCNA2		TBET			TOX		EOMES			IFNγ			SELL			IL7R	CD44
Non-proliferating Tcyt	KLRG	CD3e	CD8α	GZMB	FASL		CD69		TNFTSF9			PFN		LAG3			PDCD1			CTLA4			TIGIT	TIM3	MKI67			TOP2A			PCNA		CCNA2		TBET			TOX		EOMES			IFNγ			SELL			IL7R	CD44
TRM CD8T	ITGAE	CD3e	CD8α	GZMB	FASL		CD69		TNFRSF9			PFN		LAG3			PDCD1			CTLA4			TIGIT	TIM3	MKI67			TOP2A			PCNA		CCNA2		TBET			TOX		EOMES			ICOS			IL2RB			IL7R	ITGA1

**Table 3:** Relative abundance of immune cells in PDAC tumours 24 h after treatment. The percentages of each cell type relative to the CD45^+^ cells in the tumours of sham-exposed “control” subjects (*n* = 6), reovirus-treated group (*n* = 6), BH-treated group (*n* = 5), and the combination-treated group (*n* = 4) are shown. Percentages are shown as averages ± SEM. Statistical significance compared to controls is assumed at *p* < 0.05, and these values are highlighted in red.

Cell Abundance as a % of CD45^+^ Cells	Control (*n* = 6)	Reovirus (*n* = 6)	BH (*n* = 5)	Combination (*n* = 4)
Macrophages	12.20 ± 1.4	7.75 ± 1.3	12.21 ± 2.1	6.94 ± 1.4
Senescent Tcyt	8.30 ± 1.9	7.67 ± 1.4	4.78 ± 1.1	4.96 ± 0.9
Active neutrophils	4.86 ± 1.6	6.76 ± 2.3	10.04 ± 3.3	13.81 ± 6.5
Exhausted proliferating Tcyt	7.18 ± 2.2	7.166 ± 3.5	5.99 ± 3.03	8.49 ± 3.9
Terminal exhausted Tcyt	5.58 ± 1.2	5.41 ± 1.2	5.12 ± 0.8	3.26 ± 0.5
Active Th	6.11 ± 1.4	5.82 ± 1.2	5.08 ± 0.4	5.29 ± 1.2
Intermediate neutrophils	4.62 ± 1.0	5.12 ± 1.6	6.81 ± 1.6	8.90 ± 2.9
Inflammatory monocytes	4.59 ± 1.4	5.28 ± 0.7	5.63 ± 0.6	7.60 ± 1.8
TRM CD8T	6.04 ± 0.8	4.73 ± 1.2	4.11 ± 0.7	3.25 ± 1.1
Proliferating active Th	5.42 ± 0.6	4.82 ± 0.9	4.14 ± 0.7	3.08 ± 0.2
Tcyt subset1	4.46 ± 1.7	4.60 ± 1.8	4.71 ± 1.4	2.20 ± 1.1
B cells	3.60 ± 1.1	4.49 ± 2.0	7.25 ± 4.3	1.34 ± 0.3
Inactive neutrophils	3.96 ± 3.0	5.22 ± 4.5	2.07 ± 1.5	7.15 ± 4.1
DC	3.16 ± 0.2	3.76 ± 0.8	3.15 ± 0.7	2.67 ± 0.6
Exhausted Tcyt	2.64 ± 0.5	2.79 ± 0.6	3.11 ± 0.5	1.67 ± 0.3
Damaged cells	1.98 ± 0.5	2.39 ± 0.6	2.79 ± 0.5	2.42 ± 0.4
NK	2.00 ± 0.5	1.91 ± 0.4	2.24 ± 0.6	1.20 ± 0.2
Treg	2.29 ± 0.3	2.19 ± 0.5	1.23 ± 0.2	0.80 ± 0.2
Transitioning neutrophils	1.35 ± 0.2	1.98 ± 0.9	2.81 ± 0.6	3.03 ± 1.1
Tcyt subset2	1.72 ± 0.6	1.59 ± 0.4	1.91 ± 0.4	0.90 ± 0.3
Naïve/inactive Th	1.52 ± 0.5	1.66 ± 0.6	1.38 ± 0.2	0.87 ± 0.4
Inactive neutrophils subset	1.54 ± 1.3	1.73 ± 1.6	0.69 ± 0.6	2.07 ± 1.4
Proliferating Tcyt	1.13 ± 0.1	1.39 ± 0.2	0.72 ± 0.1	0.87 ± 0.3
MDSC-enriched	0.81 ± 0.2	0.83 ± 0.4	1.39 ± 0.5	1.46 ± 0.6
Active neutrophils subset	0.61 ± 0.5	1.02 ± 0.9	0.43 ± 0.4	3.36 ± 1.9
Intermediate neutrophils subset	0.87 ± 0.7	0.83 ± 0.8	0.23 ± 0.2	1.14 ± 0.6
B transitioning	0.80 ± 0.6	0.41 ± 0.3	0.33 ± 0.4	0.45 ± 0.4
Non-proliferating Tcyt	0.29 ± 0.2	0.27 ± 0.2	0.39 ± 0.4	0.38 ± 0.3
SIRPa^+^ cells	0.19 ± 0.1	0.18 ± 0.1	0.12 ± 0.1	0.21 ± 0.1
Tfh	0.04 ± 0.1	0.08 ± 0.1	0.03 ± 0.1	0.09 ± 0.1

**Table 4:** Ratio of immune cells to Treg 24 h after treatment. The ratios of the abundance of each immune cell type to that of Treg in the KPC tumours of each treatment group are shown as averages ± SEM. Statistical significance compared to controls is assumed at *p* < 0.05, and these values are highlighted in red.

Immune Cell Ratio to Treg	Control (*n* = 6)	Reovirus (*n* = 6)	BH (*n* = 5)	Combination (*n* = 4)
Macrophages	5.31 ± 0.61	3.52 ± 0.62	9.93 ± 1.73	8.67 ± 1.73
Senescent Tcyt	3.61 ± 0.85	3.48 ± 0.66	3.89 ± 0.89	6.20 ± 1.20
Active neutrophils	2.11 ± 0.71	3.07 ± 1.02	8.16 ± 2.67	17.27 ± 8.11
Exhausted proliferating Tcyt	3.12 ± 0.97	3.25 ± 1.57	4.87 ± 2.46	10.61 ± 4.85
Terminal exhausted Tcyt	2.42 ± 0.53	2.46 ± 0.52	4.16 ± 0.66	4.07 ± 0.61
Active Th	2.65 ± 0.59	2.64 ± 0.52	4.13 ± 0.30	6.61 ± 1.46
Intermediate neutrophils	2.01 ± 0.44	2.32 ± 0.74	5.54 ± 1.27	11.13 ± 3.60
Inflammatory monocytes	1.99 ± 0.62	2.40 ± 0.31	4.58 ± 0.49	9.50 ± 2.20
TRM CD8T	2.62 ± 0.34	2.15 ± 0.54	3.34 ± 0.57	4.06 ± 1.38
Proliferating active Th	2.35 ± 0.27	2.19 ± 0.40	3.36 ± 0.60	3.85 ± 0.23
Tcyt subset1	1.94 ± 0.72	2.09 ± 0.80	3.83 ± 1.09	2.75 ± 1.42
B cells	1.56 ± 0.49	2.04 ± 0.91	5.89 ± 3.5	1.68 ± 0.42
Inactive neutrophils	1.72 ± 1.33	2.37 ± 2.07	1.68 ± 1.23	8.94 ± 5.10
DC	1.37 ± 0.09	1.71 ± 0.34	2.56 ± 0.56	3.34 ± 0.72
Exhausted Tcyt	1.14 ± 0.28	1.27 ± 0.27	2.53 ± 0.40	2.09 ± 0.32
Damaged cells	0.86 ± 0.20	1.08 ± 0.29	2.27 ± 0.37	3.03 ± 0.52
NK	0.87 ± 0.20	0.87 ± 0.17	1.82 ± 0.49	1.50 ± 0.20
Transitioning neutrophils	0.58 ± 0.09	0.90 ± 0.41	2.28 ± 0.52	3.78 ± 1.33
Tcyt subset2	0.74 ± 0.27	0.72 ± 0.20	1.56 ± 0.30	1.12 ± 0.34
Naïve/inactive Th	0.66 ± 0.21	0.75 ± 0.26	1.12 ± 0.16	1.09 ± 0.44
Inactive neutrophils subset	0.66 ± 0.54	0.79 ± 0.72	0.56 ± 0.50	2.59 ± 1.76
Proliferating Tcyt	0.49 ± 0.05	0.63 ± 0.09	0.58 ± 0.07	1.09 ± 0.32
MDSC-enriched	0.35 ± 0.08	0.37 ± 0.16	1.13 ± 0.41	1.82 ± 0.81
Active neutrophils subset	0.26 ± 0.21	0.46 ± 0.39	0.35 ± 0.30	4.20 ± 2.43
Intermediate neutrophils subset	0.37 ± 0.30	0.37 ± 0.35	0.19 ± 0.19	1.42 ± 0.76
B transitioning	0.34 ± 0.27	0.18 ± 0.14	0.27 ± 0.31	0.56 ± 0.43
Non-proliferating Tcyt	0.12 ± 0.09	0.12 ± 0.09	0.31 ± 0.35	0.47 ± 0.31
SIRPa^+^ cells	0.08 ± 0.06	0.08 ± 0.05	0.10 ± 0.06	0.26 ± 0.12
Tfh	0.02 ± 0.01	0.04 ± 0.01	0.03 ± 0.01	0.11 ± 0.07

**Table 5:** The number of differentially expressed immune cell genes in this study. The number of genes upregulated or downregulated are shown for every immune cell subtype for comparisons of control vs. reovirus, control vs. BH, control vs. combination, combination vs. reovirus, combination vs. BH, and reovirus vs. BH. Genes were assumed to be significantly regulated if their AvgLog_2_FC expression > 1 and their *p*-adjusted value < 0.05.

Cell Clusters	Number of Genes											
	Control vs. Reovirus		Control vs. BH		Control vs. Combination		Combination vs. Reovirus		Comb. vs. BH		Reovirus vs. BH	
	Virus Up	Virus Down	BH Up	BH Down	Combination Up	Combination Down	Virus Up	Virus Down	BH Up	BH Down	BH Up	BH Down
Senescent Tcyt	2	0	0	0	3	1	1	0	0	3	1	0
Exhausted prolifer. Tcyt	18	0	30	6	15	1	1	1	16	63	153	23
Terminal exhausted Tcyt	7	0	0	0	4	1	1	0	0	0	0	0
Exhausted Tcyt	10	1	0	1	1	5	3	0	7	15	3	4
Tcyt subset1	0	0	0	0	5	2	2	2	1	2	0	0
Tcyt subset2	1	0	5	2	2	1	1	1	1	1	4	3
Proliferating Tcyt	1	0	0	0	0	0	0	0	0	0	0	0
Non-proliferating Tcyt	0	0	0	0	1	0	0	1	0	0	0	0
TRM CD8T	1	0	0	0	2	0	0	1	0	0	0	1
NK	6	0	0	0	3	6	0	2	1	0	3	1
DC	0	0	0	38	0	0	0	0	1	1	0	0
B cells	11	0	0	1	11	2	1	30	23	0	0	0
B transitioning	0	0	0	0	11	2	0	0	16	0	0	0
Naïve/inactive Th	2	1	0	0	3	0	0	0	1	1	0	0
Active Th	34	4	0	0	18	5	0	1	14	3	0	0
Proliferating active Th	32	3	0	0	34	17	0	5	12	11	1	0
Treg	2	0	0	0	1	1	1	0	0	1	0	0
Tfh	0	0	0	0	0	0	0	0	0	0	0	0
Macrophages	152	73	15	105	97	49	0	0	36	52	72	205
Inflammatory monocytes	130	94	0	0	165	34	0	1	83	43	109	205
MDSC enriched	9	4	1	0	89	17	4	31	8	4	0	0
SIRPα^+^ cells	3	0	2	0	3	0	3	0	18	3	0	0
Active neutrophils	37	1	151	17	408	217	0	1	12	1	4	11
Active neutrophils subset	0	0	1	0	1	0	0	0	0	0	0	0
Intermediate neutrophils	1	0	176	21	451	190	55	155	11	4	40	13
Intermed. Neutroph. subset	0	0	0	0	0	1	0	0	0	0	0	0
Transitioning neutrophils	15	0	9	9	131	51	2	13	10	1	0	4
Inactive neutrophils	0	0	17	1	203	325	111	58	26	49	2	1
Inactive neutrophils subset	0	0	0	0	31	44	8	15	4	0	0	0
Total number of genes differentially regulated	474	181	407	201	1693	972	194	318	301	258	392	471
	655		608		2665		512		559		863	

## Data Availability

The raw data supporting the conclusions of this article will be made available by the authors on request.

## Abbreviations

The following abbreviations are used in this manuscript:

BH	Boiling histotripsy
d.c.	Duty cycle
prf	Pulse repetition frequency
P^−^	Peak negative pressure
DC	Dendritic cells
NK	Natural killer cells
Th	CD4^+^ T helper cells
Tcyt	CD8^+^ T cytotoxic cells
MDSC	Myeloid derived suppressor cells
H&E	Haematoxylin and eosin
PDAC	Pancreatic ductal adenocarcinoma
TME	Tumour microenvironment
MHC	Major histocompatibility complex
TAP	Transporter associated with antigen processing
OV	Oncolytic virus
TRM	Tissue resident memory
SEM	Standard error of the mean
NLR	Neutrophil to lymphocyte ratio

## Appendix A

Laparotomy: Female murine subjects (aged between 12 and 20 weeks) were anaesthetised using inhalation anaesthesia (isoflurane) and were placed in a lateral recumbent position. Their fur was trimmed with a commercial animal shaver, and the surgical area was swabbed with 70% ethanol. A small abdominal incision (~5 mm) was made proximal to the spleen, the pancreas and the spleen were brought into view, and 20 μL of KPC cells were injected into the pancreas using a 29 G insulin syringe. The peritoneum was then sutured, and the skin was closed with surgical clips. Perioperative injectable analgesia was provided pre-surgery and at regular intervals up to 48 h post-surgery. Subjects were allowed to recover and monitored routinely, and 10 days post-surgery the surgical clips were removed.

Water tank and subject preparation: For boiling histotripsy (BH) (or sham) exposure the VIFU 2000 water tank was filled with warm water, degassed for a minimum of 90 min (to <2.0 mg/L of dissolved O_2_ to avoid cavitation in the water), and maintained at 34–37 °C. Before BH treatment, subjects were anaesthetised using a hypnovel/fentanyl/medetomidine/PBS (1:1:1:3) intraperitoneal injection. This was preferred to inhalation anaesthesia as it minimised respiratory body and tumour movement which would negatively impact the BH delivery. The subjects were then shaved and depilated with hair removal cream to avoid entrapment of tiny bubbles on the skin surface which could seed cavitation and result in skin damage. The anaesthetised subject was placed in the VIFU mouse holder, and secured using autoclave tape. The holder was connected to the automated VIFU gantry (Figure A1), ensuring that the head of the subject always remained above the water level, with the rest of the body immersed in the water.

Haematoxylin and eosin staining: For staining with H&E, sections were washed in xylene and then hydrated by immersion in sequentially decreasing concentrations of ethanol (100%–90%–70%). Next, the sections were stained for 10 min with Mayer’s haematoxylin, rinsed under running tap water for 5 min, and differentiated in 1% acid-alcohol for 3 s. The slides were then rinsed again under running tap water for 5 min, stained with eosin for 2 min, and washed in running tap water for another 5 min. Sections were then dehydrated in increasing concentrations of ethanol (70%–90%–100%) and washed in xylene. Before drying, slides were sealed with coverslips using an interface of DPX mounting medium. Staining visualisation was carried out using a Nanozoomer XC C12000 slide scanner driven by the Hamamatsu NDP.view2 Image viewing software (version U12388-01). This configuration facilitated automatic image acquisition of complete tumour sections at 4× magnification or snapshots taken at 20× magnification.

Tumour disintegration into single cell suspensions: Tumours were cut into approximately 3 mm × 3 mm pieces using a sterile scalpel and mixed with 2.35 mL DMEM without any supplements. To this mixture, the three proprietary enzymes provided in the Miltenyi Biotec Tumour Dissociation kit (cat. no: 130-096-730) were added and each sample was placed in a gentleMACS tube. These tubes were then placed in the gentleMACS Octo dissociator and rotated for 45 min at 37 °C. At the end of the incubation period, 10 mL of ice-cold DMEM containing 10% FBS was added to the solution. The mixture was filtered twice through 40 mm filters and washed with PBS. Cell number in the single-cell suspension was counted with a haemocytometer, and cells were stored at −80 °C in Cryostor prior to flow cytometry sorting.

Manual cluster annotation: CD4^+^ T cells: Five (5) T cell clusters overexpressed the CD4 and CTLA4 genes (Th (3 types), Treg and Tfh) (Table 2C). They all overexpressed various levels of the IL7R, TNFRSF4 [76], TIGIT, and ICOS genes. The **naïve/inactivated Th** cell cluster was differentiated from the other clusters because of its overexpression of the naïve cell CD62L (Abseq antibody) marker (Figure A2K) and the lack of the CD25/TNFRSF9 activation markers (Table 2C). Both the **non-proliferating active Th** and **proliferating active Th** cells overexpressed the CD25 and TNFRSF9 [77,78] genes, as well as the CD69, CD40LG, IFN-γ, and checkpoints LAG3 and TIM3 genes, but only the latter expressed the proliferation marker genes TOP2A [79], MKI67, and PCNA [80] (Table 2C). The **Treg** cell cluster uniquely overexpressed FOXP3 (Table 2C). A fifth proliferating cluster was identified as **Tfh** by its overexpression of the CD3G, CD4, ICOS [81], CD40LG [82], FR4 [81], IL10 [83], CTLA4, and IL7R genes, whereas no overexpression of CD69 or TNFRSF9 was seen (Table 2C).

CD8^+^ T cells: **Eight (8) Tcyt** clusters were identified as shown in Table 2D and Table A2 because these overexpressed CD3, CD8a, and Granzyme b (the latter suggesting active cytolytic activity). Memory or effector markers such as CD62L, CD44, and IL7RA were not overexpressed. Exhaustion markers such as the immune checkpoint genes PD-1, LAG-3, CTLA4, and TIGIT [84] and the activation genes FasL, TNFRSF9 [77,78], and CD69 [85] were overexpressed at various levels in each of the four clusters annotated as **senescent Tcyt**, **exhausted proliferating Tcyt**, **exhausted Tcyt**, and **terminally exhausted Tcyt** (Table 2D). The **senescent-Tcyt** cluster was annotated as such because (in addition to the markers listed above) these cells were characterised by the upregulation of the senescent marker gene KLRG1 [86]. The **exhausted-proliferating-Tcyt** cluster was characterised by the overexpression of proliferation genes including MKI67 and TOP2A, and did not overexpress the senescence marker gene KLRG1. The **terminally-exhausted Tcyt** were labelled as such because of their high levels of TIGIT gene transcription whereas the **exhausted-Tcyt** overexpressed low levels of it (Table 2D). When those two clusters were compared, the exhausted Tcyt showed increased levels of MZB and PIK3CG (Table A2). MZB has been associated with cell proliferation, inflammation, and the response to acute pancreatitis via the PI3K-Akt pathway [87], suggesting that the exhausted Tcyt may have been implicated in the response to the “chronic” damage caused by the tumours to the pancreas. The terminally exhausted Tcyt overexpressed several ribosomal proteins and AIM (Table A2). AIM has been involved in the polarisation of macrophages to a pro-tumour M2 phenotype [35]. For the remaining Tcyt clusters, the **proliferating Tcyt** were identified as such because these cells overexpressed the activation markers Granzyme B, FASL, and IFN-γ genes, and the proliferating marker genes MKI67 and TOP2A had no overexpression of the immune checkpoint genes PD-1, CTLA-4, TIM3, or TIGIT and only showed low expression levels of LAG3 (Table 2D). In contrast, the **non-proliferating Tcyt** overexpressed neither the proliferation MKI67 nor TOP2A genes (Table 2D). **Tcyt subsets 1 and 2** both overexpressed the activation marker gene CD69, as well as the immune checkpoints LAG3, PD-1, and TIGIT genes, but not CTLA-4 or proliferation markers (Table 2D). In addition to these eight Tcyt clusters, an additional CD8^+^ cluster, the **TRM CD8^+^ T cells**, was identified because the cells overexpressed the ITGAE (CD103) [88,89], ICOS, and ITGA1 (CD49b) genes (Table 2D). These cells overexpressed activation marker genes including Granzyme B, CD69, FASL, and TNFRSF9, immune checkpoint genes including LAG3, PD-1, CTLA4, and TIGIT, and the proliferation marker gene TOP2A, but were negative for CD4, therefore excluding the possibility they were TRM CD4^+^ T cells.

NK cells, DC, B cells, SIRPα^+^ cells and damaged cells: The cells in the **NK cell cluster** did not overexpress the CD3 and CD8 genes, thereby excluding the possibility they could be mistaken for Tcyt, and were positive for the NK marker gene KLRB1, the activation marker gene GZMB, as well as the NK-associated marker genes KLRK1, KLRC1, IL7R, and EOMES [90] (Table A3). **DC** were identified from their gene expression of ITGAX, the DC marker CLEC9A [91], the cross-presenting DC marker XCR1 [92], CD40, CD86, and the maturation/activation marker CD83 [93]. While the DC overexpressed the TIM3 and ITGAE [94,95] genes, they did not overexpress the monocyte/macrophage marker gene CD11b (Table A3). **B-cell** clusters were identified for their overexpression of CD19, but only one expressed the CD24a gene, which is a hallmark of transitional cells [96] (Table A3). The **SIRPα^+^ cell** cluster was annotated as such because of the positive protein expression of SIRPα on their surface (AbSeq antibody detection—Figure A2L). This cluster overlapped in the UMAP with macrophages, and the cells showed a distinct expression pattern with most of the genes being downregulated (Table A3). Hence this cluster represents a distinct set of macrophages which may have been subjected to the macrophage-inactivation effects of CD47-SIRPα signalling. Finally, the **damaged-cell** cluster was identified from its high expression of mitochondrial enzymes and overexpression of the MALAT1 gene suggestive of cells with cytoplasmic leakage (Table A3), although we cannot be certain whether our treatments, or sample processing, or a combination of both caused this phenotype (Figure A4).

Macrophages, inflammatory monocytes, and MDSC-enriched clusters: These were identified from their overexpression of typical macrophage/monocyte genes such as ADGRE1, CD68, CD86, FCGR1, FCGR3, ITGAM, ARG1, and the anti-inflammatory ITGB2 and MRC1. The S100A8 and IFITM1 genes were downregulated, and the CCL2 gene was upregulated in these two clusters separating them from neutrophils (Table A3). In addition, the **inflammatory monocytes** were defined as such because they stained positive for the Ly6C AbSeq antibody but not the Ly6G AbSeq antibody (Figure A2E). They overexpressed the CD14 and SELL genes, and the pro-inflammatory iNOS and TNFRSF1A (Table A3). In contrast, the macrophage cluster did not overexpress the genes CD14, SELL, iNOS, or TNFRSF1a, and showed high levels of ITGAX (Table A3). The **MDSC-enriched** cluster was defined as such because it stained positive for the AbSeq antibodies Ly6G and Ly6C (Figure A2E), and overexpressed the iNOS, SELL, CD33 [97], and ARG1 genes, showed low gene levels of ADGRE1, and did not overexpress ITGAX (Table A3).

## Appendix B

**Table A1:** List of AbSeq antibodies used in this study showing the antibody target and barcode sequences.

AbSeq Ab	Sequence ID	Barcode Seq
CD172a(SIRPa)	AMM2097	CGTGGTGAGTTGCGAGTGTGCGTATTATTATCTATG
CD19	AMM2007	AAGCATGTCGTTTGTGGCGTACTATTAAGGTGAAGC
CD11c	AMM2008	ATTGGGCGTAAAGGGTAAGGCGGTATATGGACTGTG
CD44	AMM2010	CATGGGTTGTCTCGTTGTAAGTAGTATAGTTGCTGC
TCR B CHN	AMM2021	CAGGTATTAGGAAGATTAGGCCGTTATGATTGGAGC
F4/80	AMM2028	GTCGTGGTCGGATAGCGTGTAGGTTTAAAGTAGAGG
H-2kb	AMM2060	CGGTATATATCTCGGAGGTAAGCGTCGCGGAAATGT
I-Ab	AMM2078	TGAGGTGTTATGTCGTTAGGGTCGTAGTGAAATTGC
Ly-6G	AMM2009	AACAATAGGGATGCGGGATAAGAATACGAAAGGAGT
Ly-6G/C	AMM2015	CATTGCGAGGAGTAAGGCGATATCTAGTTGTGCTGG
CD103	AMM2168	CAATATAATAGCCGGTAGGTGTAGTGCGTAATCAGG
CD3e	AMM2001	GAGATAGGCTAGTTGGATAATTGCGCGGTGAGAGTC
CD4	AMM2002	GTTTAGCGTAGGGTGCATTAGAGCGAGTTAGCGAGT
NK-1.1	AMM2017	GGTCTGGGATTCGTATAGTTCGCGGTAGTTGAGCTT
CD62L	AMM2018	TAGGAGAGATTCGTGGTAGATTTAGCGTAGGTCATT

**Table A2:** Differences in conserved gene expression of exhausted T cell subsets. Gene overexpression in a cluster was considered “high” if their AvgLog_2_Fc > 1 (dark green), “moderate” if 0.5 < AvgLog_2_Fc < 1 (light green), “low” if 0.25 < AvgLog_2_FC < 0.5 (amber), and “negative” (neg) if AvgLog_2_FC < 0.25 (red).

**CD8^+^ lymphocytes**										
Clusters	Additional annotation genes—Relative expression—**AvgLog_2_Fc > 1** (dark green), **0.5 < AvgLog_2_Fc < 1** (light green), **0.25 < AvgLog_2_FC < 0.5** (amber), **AvgLog_2_FC < 0.25** (red).									
Terminally Exhausted Tcyt	MZB1	DERL3	CRELD2	CDK8	PIK3CG	Ribosomal proteins	SLPI	SPP1	AIM1	TCRG.C4
Exhausted Tcyt	MZB1	DERL3	CRELD2	CDK8	PIK3CG		SLP1	SPP1	AIM1	TCRG.C4

**Table A3:** Manual annotation of NK, DC, B cells, macrophages/monocytes, damaged cells and SIRPα^+^ cells clusters observed in KPC tumours. Clusters were annotated after assessing conserved gene expression within each cluster. Gene overexpression in a cluster was considered “high” if their AvgLog_2_Fc > 1 (dark green), “moderate” if 0.5 < AvgLog_2_Fc < 1 (light green), “low” if 0.25 < AvgLog_2_FC < 0.5 (amber), and “negative” (neg) if AvgLog_2_FC < 0.25 (red).

**Natural killer, dendritic, B cells, macrophages/monocytes, damaged, MDSC and SIRPα^+^ cells**—Relative expression of annotation genes- **AvgLog_2_Fc > 1** (dark green), **0.5 < AvgLog_2_Fc < 1** (light green), **0.25 < AvgLog_2_FC < 0.5** (amber), **AvgLog_2_FC < 0.25** (red).																				
Clusters	Annotation genes																			
NK	KLRB1	CD3e	CD8a	GZMB	FASL	KLRK1	KLRC1	IL7R	TBET	EOMES										
DC	ITGAX	CLEC9A	XCR1	CD40	CD86	CD83	TIM3	CCR7	ITGAE											
B cells	CD24a	CD38	CD19	CCR7	CD69	MS4A1	IGHD	CD86	CR2	CD69	CYBB									
B transitioning	CD24a	CD38	CD19	CCR7	CD69	MS4A1	IGHD	CD86	CR2	CD69	CYBB									
Macrophages	ITGAX	ITGB2	CD86	ADGRE1	CD68	ARG1	FCGR1	FCGR3	ITGAM	CCR2	CSF1R	CYBB	CCR5	MRC1	CCL2	IFITM1	S100A8			
Inflammatory Monocytes	CD14	iNOS	SELL	TNFRSF1α	ITGB2	CD86	ADGRE1	CD68	ARG1	FCGR1	FCGR3	ITGAM	CCR2	CSF1R	CYBB	CCR5	MRC1	CCL2	IFITM1	S100A8
MDSC-enriched	CD33	CD14	iNOS	SELL	PDL1	ADGRE1	CD68	ARG1	FCGR1	FCGR3	ITGAM	CCR2	CSF1R	CYBB	RETNLG	MRC1	CCL2	IFITM1	S100A8	
Damaged cells	MALAT1	Mitochondrial genes																		
SIRPα^+^ cells	ADGRE1	CD3e	CD68	CD44	ITGAX	CD86	CD14	CD16	SELL	CD8α	ITGAL	FAS	CTLA4							

**Table A4:** Statistical significance (*p*-values) of the differences in relative cell abundance between sham-exposed controls and each treatment group (Table 3) are presented. Statistical significance is assumed at *p* < 0.05, and these values are highlighted in red.

*p*-Values (Cell Abundance)	Control vs. Reovirus	Control vs. BH	Control vs. Combination
**Macrophages**	0.019	0.997	0.019
**Senescent Tcyt**	0.759	0.110	0.158
**Active neutrophils**	0.423	0.123	0.135
**Exhausted proliferating Tcyt**	0.996	0.718	0.741
**Terminal exhausted Tcyt**	0.904	0.730	0.112
**Active Th**	0.844	0.437	0.635
**Intermediate neutrophils**	0.759	0.193	0.125
**Inflammatory monocytes**	0.604	0.470	0.174
**TRM CD8T**	0.286	0.062	0.046
**Proliferating active Th**	0.515	0.153	0.007
**Tcyt subset1**	0.945	0.896	0.273
**B cells**	0.647	0.344	0.095
**Inactive neutrophils**	0.784	0.546	0.497
**DC**	0.366	0.991	0.365
**Exhausted Tcyt**	0.833	0.520	0.199
**Damaged cells**	0.546	0.188	0.470
**NK**	0.855	0.728	0.147
**Treg**	0.823	0.016	0.004
**Transitioning neutrophils**	0.426	0.027	0.089
**Tcyt subset2**	0.838	0.763	0.258
**Naïve/inactive Th**	0.819	0.769	0.285
**Inactive neutrophils subset**	0.907	0.513	0.758
**Proliferating Tcyt**	0.225	0.016	0.310
**MDSC-enriched**	0.951	0.220	0.268
**Active neutrophils subset**	0.625	0.750	0.127
**Intermediate neutrophils subset**	0.967	0.356	0.759
**B transitioning**	0.515	0.483	0.629
**Non-proliferating Tcyt**	0.939	0.813	0.771
**SIRPa^+^ cells**	0.947	0.632	0.928
**Tfh**	0.221	0.720	0.371

**Table A5:** Statistical significance of the differences in the immune cell/Treg ratio between sham-exposed controls and each treatment group (Table 4) are presented. Statistical significance is assumed at *p* < 0.05, and these values are highlighted in red.

*p*-Values (Cell Ratios over Treg)	Control vs. Reovirus	Control vs. BH	Control vs. Combination
**Macrophages**	0.031	0.0132	0.056
**Senescent Tcyt**	0.892	0.798	0.079
**Active neutrophils**	0.368	0.025	0.046
**Exhausted proliferating Tcyt**	0.930	0.441	0.095
**Terminal exhausted Tcyt**	0.957	0.038	0.053
**Active Th**	0.984	0.034	0.016
**Intermediate neutrophils**	0.664	0.010	0.013
**Inflammatory monocytes**	0.491	0.004	0.003
**TRM CD8T**	0.383	0.231	0.244
**Proliferating active Th**	0.688	0.097	0.002
**Tcyt subset1**	0.866	0.119	0.560
**B cells**	0.587	0.168	0.850
**Inactive neutrophils**	0.753	0.979	0.128
**DC**	0.27	0.030	0.009
**Exhausted Tcyt**	0.709	0.008	0.039
**Damaged cells**	0.461	0.003	0.001
**NK**	0.990	0.058	0.041
**Transitioning neutrophils**	0.385	0.003	0.016
**Tcyt subset2**	0.928	0.043	0.361
**Naïve/inactive Th**	0.735	0.074	0.317
**Inactive neutrophils subset**	0.874	0.876	0.234
**Proliferating Tcyt**	0.152	0.252	0.049
**MDSC-enriched**	0.866	0.047	0.051
**Active neutrophils subset**	0.600	0.780	0.076
**Intermediate neutrophils subset**	0.997	0.566	0.160
**B transitioning**	0.539	0.833	0.631
**Non-proliferating Tcyt**	0.978	0.536	0.219
**SIRPa^+^ cells**	0.991	0.841	0.161
**Tfh**	0.188	0.554	0.120
